# Multi-site assessment of microtidal wave-dominated active beach state and morpho-sedimentary parameters using optical satellite imagery

**DOI:** 10.1038/s41598-026-45638-3

**Published:** 2026-03-27

**Authors:** Salomé Frugier, Rafael Almar, Erwin W. J. Bergsma, Marcan Graffin, Gerben Ruessink

**Affiliations:** 1 LEGOS (Laboratoire d’Etudes en Géophysique et Océanographie Spatiales), Toulouse, France; 2 CNES (Centre National d’Etudes Spatiales), Toulouse, France; 3 Department of Physical Geography, Faculty of Geosciences, Utrecht University, Utrecht, The Netherlands

**Keywords:** Climate sciences, Environmental sciences, Natural hazards, Ocean sciences, Solid Earth sciences

## Abstract

Traditionally, beach states are defined from visual observations, in-situ measurements and/or video imagery, which limits their application to a handful of well-instrumented sites. In this work, we propose a different approach by focusing on a remotely observable quantity: the cross-shore distance between the offshore wave-breaking and the shoreline position, denoted $X_b$. This metric defines the *active* beach state, capturing where waves dissipate energy relative to the underlying morphology. Using 10 years of Sentinel-2 imagery, $X_b$ is evaluated across 30 wave-dominated microtidal sandy beaches spanning reflective to fully dissipative conditions. The metric reproduces the structure of classical beach state frameworks and enables classification into five active states (R, LTT, TBR/RBB, LBT, and D) using transferable thresholds. $X_b$ is continuous, thus it also reveals how beach state evolve through time, allowing quantification of state occurrence, residence time, and transitions, with seasonal variability consistent with independent classifications at well-studied sites. Furthermore, using empirical relationships, we demonstrate that $X_b$ carries first-order information about beach-face slope ($\tan \beta$) and sediment grain size ($D_{50}$), opening a pathway toward systematic satellite-based monitoring of coastal morphodynamics at regional to global scales.

## Introduction

Beach morphodynamics describes the dynamic interaction between beach morphology and hydrodynamic forcing^1^. Wave transformation across the nearshore area - including shoaling, surf, and swash processes - drives sediment transport patterns that continuously reshape the beach profile^2–4^. These hydro-sedimentary processes operate over a wide range of temporal scales, from storm-driven events^5^ to seasonal^6^ and interannual variability^7–9^, and are modulated by environmental settings such as wave climate, sediment supply, and tidal regime^10^.

To capture this temporal and spatial variability, Wright and Short (1984)^11^ introduced the concept of beach state, describing six morphodynamic states along a reflective-dissipative continuum, with four intermediate states described mainly by nearshore sandbar configurations (e.g. parallel, rhythmic, transverse, or terrace). Since then, the beach state classification has been further refined and extended, notably by Masselink and Short (1993)^12^, who incorporated the role of tidal range in beach state classification, and more recently by Castelle and Masselink (2023)^1^, who proposed updated morphodynamic classifications. Beach states classify nearshore morphodynamic configurations according to their hydrodynamic forcing, providing an integrated description of wave conditions, beach slope, and sediment characteristics. Inversely, one can use the beach state to get a qualitative idea of the local hydro-morphodynamic parameters^13,14^. Dimensionless parameters such as the fall velocity parameter^11,15^, as well as the Iribarren number^16,17^, link hydrodynamic forcing to morphological characteristics through sediment grain size and beach slope. While these approaches provide a physically grounded framework, their application requires in-situ measurements of parameters such as breaking wave height, sediment grain size, or beach slope, which remain unknown for most of the world’s coastlines.

Prior to the classification of Wright and Short (1984)^11^, beach states had already been investigated in New South Wales (NSW), Australia. Based on daily visual observations at Narrabeen Beach, Short (1979)^18^ emphasized *temporal* variability by exploring transitions across the reflective-dissipative spectrum and identifying erosional versus accretionary phases. At the same time, Wright et al. (1979)^19^ highlighted the importance of *spatial* variability by comparing multiple beaches through wave measurement surveys. Wright and Short (1984)^11^ then provided the link between beach states and physically based parameters derived from direct in-situ measurements. Even though the use of direct measurements reduced the level of subjectivity, it was still limited by the local requirements of the data.

The development of video monitoring systems in the 1980 s marked a step forward by shifting focus localized in-situ measurements toward spatially coherent wave-breaking patterns. Lippmann and Holman (1989)^20^ were the first to exploit video imagery to track sandbar positions through time-averaged sequences (time-exposures), attempting also a more objective beach state classification^21^. However, all beach states were identified visually, thereby still introducing some level of subjectivity to image classification. ARGUS-type video stations facilitated near-continuous observations of wave-breaking patterns, which were further used in multiple studies to infer sandbar dynamics (e.g^22–25^.,). Ranasinghe et al. (2004)^26^ introduced a more objective method that combined video imagery with quantitative metrics - surf zone width and alongshore morphological variability - to distinguish between beach types. In recent years, the emergence of deep learning has further advanced this line of work. In fact, several studies have used Convolutional Neural Network (CNN) to automate beach state classification from video imagery, including for both single-^27^ and double-bar systems^28^. Despite these advances, all these approaches remain site-dependent, as they rely on either in-situ surveys or fixed video systems. Consequently, beach state variability along extended coastlines or even within the same coastal sector remains difficult to observe at large spatial scales.

Satellite remote sensing provides an opportunity to define and classify beach states, revealing how they vary in space and time, and their distribution at large spatial scales. Optical satellites provide repeated and long-term global observations of the coastal environment, making them well suited for coastal change monitoring^29^. Much of the existing satellite-based work has therefore focused on shoreline extraction from multispectral satellite imagery. Numerous algorithms exist for land-water interface detection based on spectral indices and thresholding methods (e.g., CASSIE^30^, CoastSat^31^, SHOREX^32^, ShorelineMonitor^33^, High-TideSDS^34^, Shoreliner^35^), which have enabled the creation of global shoreline datasets with continuous, albeit coarse, coverage^9,33^. In contrast, more three dimensional analyses remain experimental^36^ or the coupling to underwater features like satellite-based detection of nearshore sandbars, remains limited. Although sandbars have been investigated using video imagery and local surveys, there is a lack of automated and transferable satellite methods^37–39^. Consequently, characterising surf-zone organisation and beach states across extended coastlines remains challenging, with almost no large-scale examples except for Aleman et al. (2015)^40^, who analysed $\sim$200 km of coastline using topo-bathymetric LiDAR in a low-energy storm-influenced nontidal environment.

Rather than relying on environmental parameters that are difficult to measure consistently at large spatiotemporal scales, this study focuses on a directly observable quantity: the cross-shore distance between the offshore wave-breaking position and the shoreline, denoted $X_b$. We introduce the concept of an *active* beach state, emphasizing the wave-driven configuration of the nearshore rather than its static morphology. We investigate whether $X_b$ can provide a scalable proxy for beach state classification and whether it retains information about key morpho-sedimentary parameters.

Being able to observe beach state variability across regions is not only a matter of methodology. Morphodynamic configurations influence beach safety^41,42^, as three-dimensional bar systems are associated with rip current activity^43^, a major hazard to swimmers. Sandbar configuration also has an impact for the suitability of beaches for boat landings, historically of strategic importance, for example during the Normandy landings in 1944^44^. Moreover, sandbars act as dynamic natural buffers, controlling wave breaking and energy dissipation across the nearshore area. Through this modulation of wave energy, resulting morphodynamic states affect shoreline stability and thus exposure to erosion hazards with major societal and economic consequences^45^, while also shaping coastal attractiveness and recreational use^46^. A scalable approach to beach state classification therefore provides a pathway to better understand and compare coastal vulnerability across regions.

## Methods

### Study sites

**Fig. 1:**
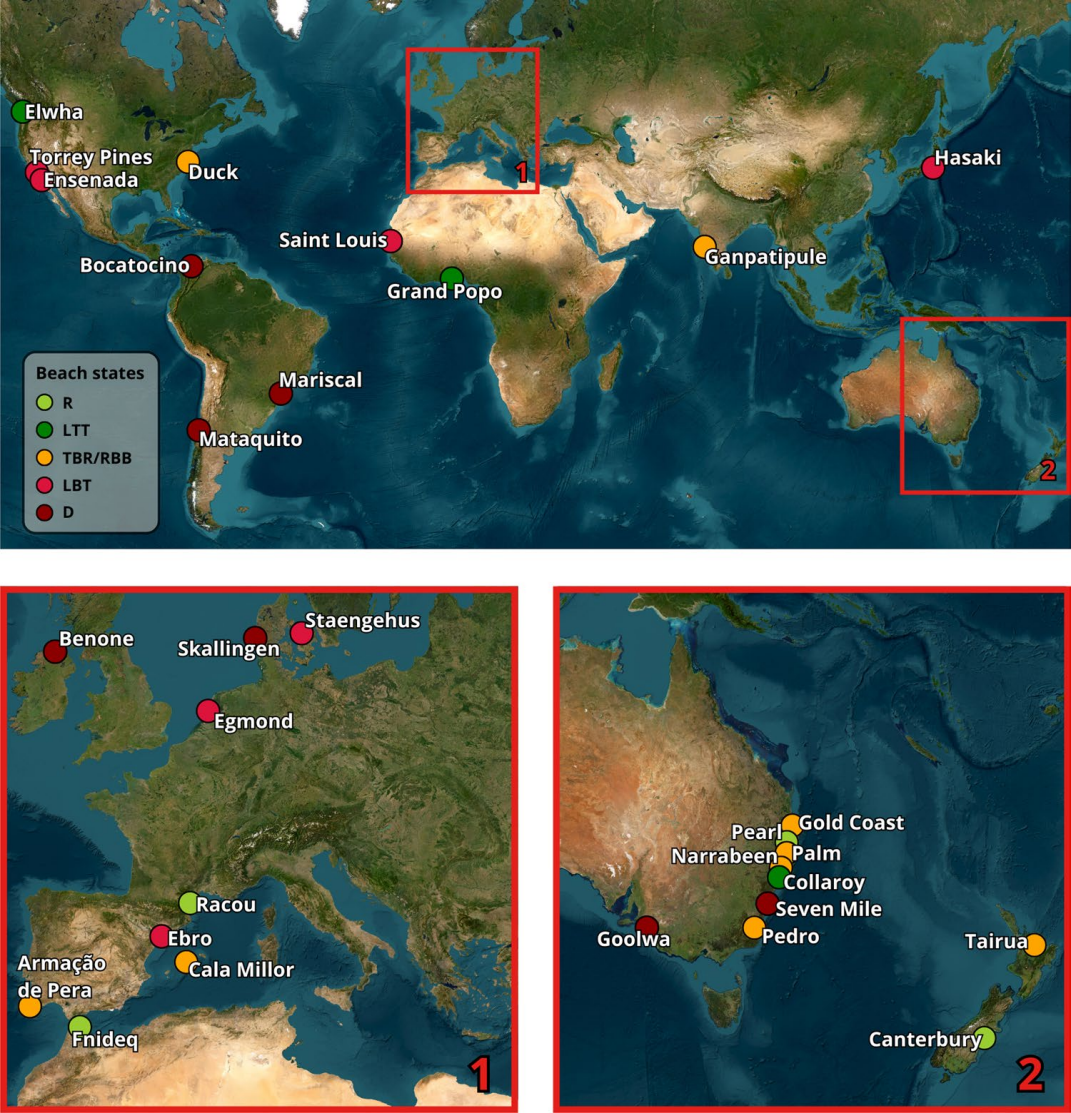
Distribution of the 30 reference beach sites used in this study. Sites are distributed across the five continents. Marker color represent the classified median beach state from literature: light green for R, dark green for LTT, orange for TBR/RBB, red for LBT, and dark red for D.

This study uses a dataset of 30 well-documented microtidal wave-dominated beach sites distributed across the five continents (Figure 1). These sites unfold a wide range of morphodynamic beach states and physical characteristics, providing a robust basis for validation (Table 1).

Reflective (R) beaches are often referred to as ”fully accreted/summer profiles” due to their steep profiles^18^. The absence of a bar confines the breaking process to the upper runup zone on the beach face (surging or collapsing breaker types). As a result, the refraction coefficient is low and between 60$\%$ and 80$\%$ of the wave energy is reflected^17,47^. Different types of reflective beach states were selected, typically featuring coarse sediment and steep slopes. The Racou beach in South France^40^ and Pearl in Australia^48^ are pocket beaches, while Canterbury^49^ and Fnideq^50^ are open coast beaches.

Intermediate beach states exhibit the most complex morphological setting, as well as the most complex processes. There is a coexistence of dissipative and reflective features, meaning that energy dissipation varies both cross-shore and longshore. Low Tide Terrace (LTT) beaches in microtidal environments have a steep slope on the beach face followed by a terrace resembling a dissipative profile. However, the breakers generally occur slightly offshore compared to reflective beaches, due to the presence of this terrace. The LTT state sites chosen are Grand Popo in Benin^51^, Collaroy in Australia^52^ and Elwha^53^ in United States (US). The Rythmic Bar and Beach (RBB) and Transverse Bar and Rip (TBR) morphologies show more longshore variability in bar shape than Longshore Bar and Trough (LBT) or LTT states, mainly due to the presence of rip channels^26^. In this study, RBB and TBR are grouped together. In fact, the resolution of satellite images (10-m for Sentinel-2) and the instantaneous nature of the satellite imagery make it difficult to distinguish between RBB and TBR states (discussed in Section Limitations and Perspectives). Even in the literature, the distinction between these two beach types is rarely clearly made; they are often described as rhythmic/crescentic beaches or rip-channel/transverse bar beaches^18^. Four beaches in Australia were selected: Pedro^54^, Gold Coast^55^, Narrabeen^52^, and Palm Beach^26^. Beaches with differing characteristics were selected: for example, Gold Coast features a double-bar system with net offshore migration (NOM)^23^, whereas Pedro maintains an TBR/RBB state over time. In addition to the Australian beaches, four more sites were selected from the literature: Duck (US)^21^, Tairua (New Zealand)^56^, Cala Millor (Spain)^57^ and Armação De Pera (Portugal)^58^. One additional site, Ganpatipule (India), lacks a clearly reported dominant beach state in the literature; here it is classified as TBR/RBB based on hydro-morphodynamic parameters ($D_{50}$, slope, and wave height) together with visual inspection of the full satellite image time series^59^.

While crescentic bar structures are typically associated with low-frequency wave regimes under non-storm conditions^21^, the formation of LBT systems occurs during highly energetic wave regimes. Sediment redistribution in both the cross-shore and longshore directions drives the morphological evolution of LBT systems^60^. For this beach state, sites such as Egmond aan Zee in the Netherlands^61^, one beach close to the Ebro Delta in Spain^60^, Saint-Louis in Senegal^62^, and Staengehus in Denmark^48^, which feature multi-bar systems where the inner bar can take on rhythmic longshore forms are selected. Torrey Pines in California (US) and Ensenada in Baja California (Mexico) are transitional sites between microtidal and mesotidal conditions (RTR<3, with RTR being the Relative Tidal Range^12^), where the single offshore bar responds to seasonal wave cycles^63,64^. The Hasaki site in Japan also exhibits a similar behavior, with NOM of bars occurring with an average of one year-periodicity^65^.

Unlike the LBT state, where broken waves cease to dissipate after crossing the steep inner edge of the bar and reform in the deep trough, dissipative (D) states exhibit strong and continuous energy dissipation from the first breaking point onward, without significant wave reformation, maximizing dissipation across the surf zone. Beaches with varying degrees of dissipation were selected, including Goolwa in Australia, which is known as a fully dissipative beach with fine sand, a low gradient, and a wide multi-bar surf zone with no 3D longshore rhythmicity. Waves break in a spilling pattern, dissipating energy gradually across the broad surf zone before becoming very small at the beach face^66,67^. Mataquito in Chile is another highly dissipative beach with an energetic wave regime^68^. Bocatocino in Colombia^69^, Benone in Ireland^70^, Seven Mile in Australia^67^, Skallingen in Denmark^48^, and Mariscal in Brazil^71^ are beaches where the most common state is dissipative, though they can also transition into other states.

**Table 1:** Summary of site-specific morphodynamic and hydrodynamic characteristics for the 30 validation beaches. $H_s$ and $T_p$ are representative annual average significant wave height and peak period values compiled from the literature. The corresponding source locations (coordinates and depth, when available) are provided in Table **S2** in the Supplementary material.

Site	Country	Beach state	Hs (m)	Tp (s)	Slope	$D_{50}$ ($\mu$m)	TR (m)	$\Omega _b$	$\xi$	RTR	$\Delta x$ (m)
Canterbury^49,72^	New Zealand	R	1	5.5	0.105	460	1.5	2.6	0.6	1.2	14
Fnideq^50^	Morocco	R	0.5	3.5	0.09	1100	1.0	1.7	0.6	1.3	11
Racou^40^	France	R	0.3	6.0	0.05	912	0.3	1.1	0.7	0.4	6
Pearl^48^	Australia	R	0.25	8.5	0.07	300	1.6	1.4	1.5	1.9	23
Grand Popo^51^	Benin	LTT	1.36	9.4	0.10	600	1.8	1.9	1.0	1.05	18
Elwha^53^	US	LTT	0.47	6.5	0.12	4200	1.4	0.6	1.4	1.5	12
Collaroy^52^	Australia	LTT	1.6	10.0	0.10	500	1.3	2.1	1.0	0.7	13
Narrabeen^52^	Australia	TBR-RBB	1.6	10.0	0.10	355	1.3	2.5	1.0	0.7	13
Gold Coast^23,55^	Australia	TBR-RBB	0.8	9.5	0.02	250	2.0	2.3	0.3	1.4	100
Duck^21^	US	TBR-RBB	0.9	8.3	0.08	150	1.2	3.4	0.9	0.9	15
Pedro^54^	Australia	TBR-RBB	1.4	9.0	0.08	350	1.3	2.6	0.8	0.8	16
Tairua^56^	New Zealand	TBR-RBB	1.6	7.4	0.105	600	2.0	2.3	0.8	1.2	19
Palm^26^	Australia	TBR-RBB	1.5	7.0	0.03	300	1.6	3.3	0.2	1.0	53
Ganpatipule^59^	India	TBR-RBB	1.5	5.5	0.025	400	2.0	3.1	0.1	1.5	80
Armaçao de Pera^58^	Portugal	TBR-RBB	1.0	5.0	0.085	450	2.0	2.8	0.5	1.7	24
Cala Millor^57^	Spain	TBR-RBB	0.52	6.15	0.044	200	0.2	2.9	0.5	0.2	5
Hasaki^65^	Japan	LBT	1.35	8.0	0.025	180	1.4	3.8	0.2	0.9	56
Saint Louis^62^	Senegal	LBT	1.52	9.23	0.048	210	1.6	3.4	0.5	0.9	33
Egmond aan Zee^61^	Netherlands	LBT	1.2	5.0	0.0135	300	2.1	3.7	0.1	1.7	156
Torrey Pines^63^	US	LBT	1.1	12.0	0.04	230	2.3	2.4	0.6	1.3	56
Ensenada^64^	Mexico	LBT	1.0	11.0	0.03	250	2.3	2.3	0.4	1.4	77
Staengehus^48^	Denmark	LBT	2.5	6.5	0.016	250	0.2	4.6	0.1	0.1	13
Ebro^60^	Spain	LBT	0.7	5.0	0.035	260	0.2	3.2	0.3	0.2	6
Goolwa^66,67,73^	Australia	D	3.0	10.0	0.011	120	0.8	5.6	0.1	0.3	73
Seven Mile^67^	Australia	D	2.0	10.0	0.015	180	1.6	3.9	0.1	0.8	107
Bocatocino^69,74^	Colombia	D	1.7	7.0	0.014	125	0.5	5.4	0.1	0.3	34
Mariscal^71^	Brazil	D	0.5	8.0	0.05	30	0.8	6.2	0.7	0.7	15
Skallingen^48,75^	Denmark	D	1.1	4.0	0.025	175	1.8	5.3	0.1	1.6	72
Benone^70^	Ireland	D	2.05	6.9	0.0194	157	1.6	5.2	0.1	0.9	82
Mataquito^68^	Chile	D	2.4	10.0	0.06	250	1.4	3.5	0.5	0.7	24

### Satellite and wave datasets

Sentinel-2 MultiSpectral Instrument (MSI) Level-1C Top-Of-Atmosphere (TOA) reflectance images were retrieved for all 30 sites using the Google Earth Engine (GEE) Python API^76,77^. The Sentinel-2 MSI measures reflected solar radiance, which is converted to TOA in Level-1C products and do not depend on the choice of atmospheric correction routine, ensuring methodological consistency across sites and over time. Images spanning June 2015 to March 2025 were requested. Sentinel-2 provides a spatial resolution of 10 m for visible and near-infrared bands and a nominal revisit time of 5 days. The blue (B), green (G), red (R), near-infrared (NIR), and short-wave infrared (SWIR1 and SWIR2) bands were used. SWIR1 and SWIR2, originally at 20 m resolution, were resampled to 10 m using bi-cubic interpolation to ensure spatial consistency with the other spectral bands. A cloud-cover threshold was first applied using GEE built-in metadata. Because this threshold is defined at tile scale and may reject images that are cloud-free over the Region of Interest (ROI), all scenes with total cloud cover below 90% were retained. A second filtering step was then applied at ROI scale using the method of Graffin et al. (2025)^8^, which detects cloud contamination based on the pixel-scale distribution of the Subtractive Coastal Water Index (SCoWI; see Methods [3] for the definition of SCoWI and Figure **S1** in Supplementary material for an example of cloud image filtering).

Offshore wave conditions were obtained from the ERA5 reanalysis dataset^78^. For each site, the closest ERA5 wave model grid node to the ROI was selected. Significant wave height ($H_s$, m) and peak wave period ($T_p$, s) were extracted over the same time window. For each satellite acquisition, ERA5 values were sampled at the hour closest to the satellite overpass time.

### Shoreline and breaking detection

The shoreline proxy corresponds to the waterline, meaning the instantaneous land-water interface at the time of satellite image acquisition^79^. This proxy reflects variations in sea level, including tidal fluctuations, which can induce a cross-shore excursion of the waterline when no tidal correction is applied. A first-order estimate of the maximum cross-shore waterline displacement, approximated as $\Delta x = TR/\tan \beta$, ranges from 6 to 156 m across the study sites and remains below 20 m for 15 sites (Table 1, with *TR* the Tidal Range (m) and $\tan \beta$ the slope). This indicates that, while the induced variability is limited for most steep, microtidal beaches, it can become substantial for gently sloping environments. The implications of tidal modulation are further discussed in Section Limitations and Perspectives. The shoreline was extracted using the method developed by Bergsma et al. (2024)^35^, specifically designed and validated for sandy environments. This approach relies on the SCoWI index, combined with modified Otsu thresholding, referred to as Local Minimum thresholding^80^ and the Marching Squares algorithm to derive sub-pixel shoreline positions^81^:

1 $$\begin{aligned} \text {SCoWI} = \text {B} + 2(\text {G} - \text {NIR}) - 0.75\,\text {SWIR1} - 0.5\,\text {SWIR2} \end{aligned}$$

where B, G, NIR, SWIR1 and SWIR2 denote TOA values of the blue, green, near-infrared and short-wave infrared bands.

Wave-breaking zones were identified using the Normalized SandBar Index (NSBI), derived from the Sandbar Index (SBI)^39^:

2 $$\begin{aligned} \text {SBI} = 2(\text {B} - \text {R}) + \text {G} + 0.25\,\text {NIR} \end{aligned}$$

3 $$\begin{aligned} \text {NSBI} = \frac{\text {SBI} - \text {SBI}_{min}}{\text {SBI}_{90} - \text {SBI}_{min}} \end{aligned}$$

where $\text {SBI}_{90}$ represents the 90th percentile of SBI values within the ROI. The use of the 90th percentile rather than the absolute maximum reduces sensitivity to extreme reflectance values caused by specular reflections or sensor noise. Normalization ensures comparability across different satellite missions (e.g., Landsat, VEN$\mu$S, Sentinel-2). The NSBI formulation and associated threshold (NSBI > 1.2) were optimised across multiple sandy sites to maximise discrimination between breaking and non-breaking pixels (land, sand and water). The selected threshold was independently validated at Duck (NC, US)^82^, where regular in-situ bathymetric surveys allow direct comparison between satellite-derived breaking patterns and observed sandbar morphology.

### Analysis domain and transects

The spatial configuration adopted for each site is summarized in Table **S3** and illustrated schematically in Figure **S4** in Supplementary material. For each beach, the analysis domain corresponds to an alongshore segment bounded by two endpoints, A and B, separated by a an alongshore shoreline distance *L*. Points A and B define the land origins of the first ($\textrm{TR}_0$) and last ($\textrm{TR}_n$) cross-shore transects, respectively. By convention, point A corresponds to the endpoint with the largest latitude. A baseline was digitized along the landward edge of the beach in *QGIS*^83^, following either the vegetation line when clearly identifiable or, otherwise, a line conforming to the local shoreline geometry (e.g., rectilinear or curved). Cross-shore transects were then generated perpendicular to this baseline and extended seaward over a distance $\ell$, ensuring full coverage of the surf zone and offshore breaking region. Transects were spaced at regular intervals of $e = 30$ m, yielding a total of *n* transects per site. Because morphodynamic state can vary substantially alongshore, the spatial domain was selected carefully for each beach. For long rectilinear coasts, a representative sector ($L\sim 2km$) was analysed rather than the full shoreline extent. For embayed beaches, transects were restricted to the most morphodynamically coherent section, avoiding sheltered or shadowed areas with distinct wave exposure. In the case of Narrabeen, where documented alongshore contrasts exist, separate sectors were analysed independently to prevent mixing different morphodynamic configurations (Narrabeen = TBR/RBB, Collaroy = LTT^52^).

### Extraction of shoreline and breaking position

For each acquired satellite image, the extracted shoreline was intersected with transects to obtain the cross-shore shoreline position $x_s$ (m), defined as the cross-shore distance from the transect origin. This shoreline proxy extraction approach has been previously validated against in-situ observations across multiple sites^84^. Offshore wave-breaking was mapped using the NSBI threshold (NSBI > 1.2) and similarly intersected with the transects to obtain breaking positions $x_b$ (m). Because wave breaking is instantaneous and spatially heterogeneous, $x_b$ can vary substantially alongshore within a single image. For example, on single-bar beaches, some transects may exhibit breaking on the bar while others show no breaking (case of three dimensional bar systems); on double-bar beaches, breaking may occur on either the inner or outer bar depending on local conditions. To consistently identify the offshore breaking associated with the dominant terrace/bar system, the set of $x_b$ values for each image was clustered along the cross-shore axis using a K-means algorithm. Cluster quality was evaluated using the silhouette score, and only well-separated clusters (silhouette > 0.65) were retained. When clustering was reliable, the most offshore cluster was selected. If clustering was not robust, a single breaking line position was assumed. To avoid selecting isolated breaking events unrelated to the morphodynamic system, a minimum fraction of transects exhibiting breaking was required: clusters with fewer than 20% of transects containing valid breaking detections were discarded. K-means was performed using a fixed number of clusters ($k = 2$), reflecting the typical occurrence of one or two dominant breaking lines (inner and outer bar systems). Although additional sandbars may be present, simultaneous breaking rarely occurs on more than two major bars, except under fully dissipative conditions.

### $X_b$ time series and signal processing

For each satellite acquisition, the active beach state proxy was defined as:

4 $$\begin{aligned} X_b = x_b - x_s \end{aligned}$$

where $x_b$ (m) and $x_s$ (m) are the cross-shore offshore breaking and shoreline positions, respectively, both measured along each transect from the landward origin. For a given image, $X_b$ was computed for all valid transects and aggregated using the median to obtain a representative cross-shore breaking distance for the site. The standard deviation was also calculated to quantify alongshore variability. Outliers in the $X_b$ time series were identified using the Inter-quartile Range (IQR) method^84^. The IQR was defined as the difference between the third quartile (Q3) and the first quartile (Q1), and observations falling outside 1.5 $\times$ IQR were removed. Two complementary signals were then defined. The *instantaneous signal* which correspond to the raw $X_b$ values (individual satellite acquisitions), representing the short-term response of the beach system to hydrodynamic forcing (waves and tides). To characterize longer-term variability, a *filtered signal* was constructed. The $X_b$ time series was first resampled to 30-day intervals. Short gaps were filled using cubic spline interpolation, while gaps exceeding 90 days were left as missing to avoid artificial smoothing. A 3-month rolling average was applied to reduce short-term fluctuations and highlight the underlying seasonal to inter-seasonal variability. The raw time series exhibited substantial high-frequency variability associated with individual storm events, which obscured longer-term trends relevant to morphodynamic state transitions (see Section Beach state variability and stability). Because Sentinel-2A operated alone between 2015 and 2018, image availability during this early period was too sparse to allow consistent computation of the 3-month moving average. Therefore, analyses involving the *filtered signal* were restricted to the 2018-2025 period, when both Sentinel-2A and 2B were operational and temporal sampling was sufficient.

### Temporal coverage and sampling characteristics

The temporal coverage of the $X_b$ time series varies slightly among sites due to data availability, wave-breaking conditions, and cloud cover. For each site, the start and end dates of the $X_b$ time series are reported in Table **S1** in the Supplementary Material. Overall potential observation window spans approximately June 2015 to February 2025.

To further assess sampling robustness, we analyzed (i) the seasonal distribution of breaking image acquisitions (DJF, MAM, JJA, SON) and (ii) the temporal resolution of the $X_b$ time series. Because $X_b$ can only be derived from images exhibiting detectable wave breaking, the temporal resolution directly reflects the availability of breaking observations. These two components are respectively presented in Figure **S4(a)** (seasonal distribution) and **S4(b)** (temporal resolution) in the Supplementary Material. Temporal resolution was quantified as the distribution of time intervals ($\Delta t$) between consecutive valid $X_b$ observations for each site. To ensure consistent sampling conditions under dual Sentinel-2A/2B operation, this analysis was restricted to the *instantaneous signal* during 2018-2025 period. Clear regional patterns emerge. Mediterranean (MED) sites exhibit the lowest number of breaking images, resulting in the poorest temporal resolution, with a mean $\Delta t$ of approximately 40 days. This reduced sampling primarily reflects their low-energy wave climate, which generates fewer detectable breaking events. Northern Europe (NE) follows, with a mean $\Delta t$ of about 21 days, where the reduced temporal resolution is mainly associated with persistent cloud cover limiting usable observations. In contrast, sites located in North America, South America, Africa, Asia and Oceania display a much higher sampling density, with a mean $\Delta t$ of approximately 9 days, indicating a strong temporal resolution driven by frequent breaking conditions. Seasonal patterns further reflect hemispheric variability. In Northern Hemisphere sites (from Staengehus to Bocatocino), fewer breaking images are observed during boreal summer (JJA). In contrast, Southern Hemisphere sites (from Mariscal to Canterbury) show increased breaking-image availability during JJA, corresponding to austral winter conditions and enhanced wave activity.

### Linking $X_b$ to morpho-sedimentary parameters

Reference morphodynamic parameters ($H_s$, m; $T_p$, s; $D_{50}$, m; $\tan \beta$) were compiled from the literature for each site (Table 1). To investigate whether the *active* beach state proxy $X_b$ retains information about underlying morpho-sedimentary parameters, we examined its relationship with two classical dimensionless parameters used in beach state theory: the fall velocity parameter $\Omega _b$^11,15^ and the Iribarren number $\xi$^16,17^. For each satellite acquisition, offshore wave conditions ($H_s$, $T_p$) from ERA5 were used to approximate the breaking wave height $H_b$ following Komar (1974) (Eq. 5). The fall velocity parameter $\Omega _b$ was then computed from $H_b$, $T_p$, and the sediment fall velocity $w_s$ (Eq. 6), with $w_s$ expressed as a function of $D_{50}$ (see definition below Eq. 6). The Iribarren number $\xi$ was computed from beach slope $\tan \beta$, offshore wave height $H_s$, and deep-water wavelength *L* (Eq. 7). Empirical relationships between median $X_b$ and these dimensionless parameters were explored using several candidate functional forms (linear, logarithmic, exponential, and power-law models). For each parameter, the best-fit relation was selected based on its consistency with both the dataset and the expected physical behavior. A power-law relation provided the most robust fit between $X_b$ and $\Omega _b$ (Eq. 8), while an exponential decay described the relationship between $X_b$ and $\xi$ (Eq. 9). Substituting Eqs. 8 and 9 into the definitions of $\Omega _b$ and $\xi$ (Eqs. 6 and 7) allowed inversion toward first-order estimates of sediment grain size $D_{50}$ and beach slope $\tan \beta$, expressed solely as functions of $H_s$, $T_p$, and $X_b$ (Eqs. 10 and 11). These formulations provide empirical first-order morpho-sedimentary proxies derived from remotely sensed wave-breaking extend, enabling large-scale estimation in regions where in-situ measurements are unavailable.

5 $$\begin{aligned} H_b = 0.39\, g^{1/5}(H_s T_p)^{2/5} \end{aligned}$$

6 $$\begin{aligned} \Omega _b = \frac{H_b}{T_p w_s} \end{aligned}$$

where $w_s = \sqrt{(s-1)gD_{50}}$ ($m s^{-1}$) is the sediment fall velocity, $s=2.58$ the relative sediment density, and $g=9.81$ $m s^{-2}$ gravitational acceleration.

7 $$\begin{aligned} \xi = \frac{\tan \beta }{\sqrt{H_s/L}} \end{aligned}$$

with $L = \frac{gT_p^2}{2\pi }$ (m) the deep-water wavelength.

8 $$\begin{aligned} \Omega _b = 0.50\, X_b^{0.43} \end{aligned}$$

9 $$\begin{aligned} \xi = 1.4\, e^{-0.02 X_b} + 0.07 \end{aligned}$$

10 $$\begin{aligned} D_{50} = \frac{1}{(s-1)g} \left( \frac{0.39\, g^{1/5}(H_s T_p)^{2/5}}{T_p(0.50X_b^{0.43})} \right) ^2 \end{aligned}$$

11 $$\begin{aligned} \tan \beta = (1.4 e^{-0.02X_b}+0.07) \sqrt{\frac{2\pi H_s}{gT_p^2}} \end{aligned}$$

## Results and discussion

### Satellite-derived proxy for beach state classification

**Fig. 2:**
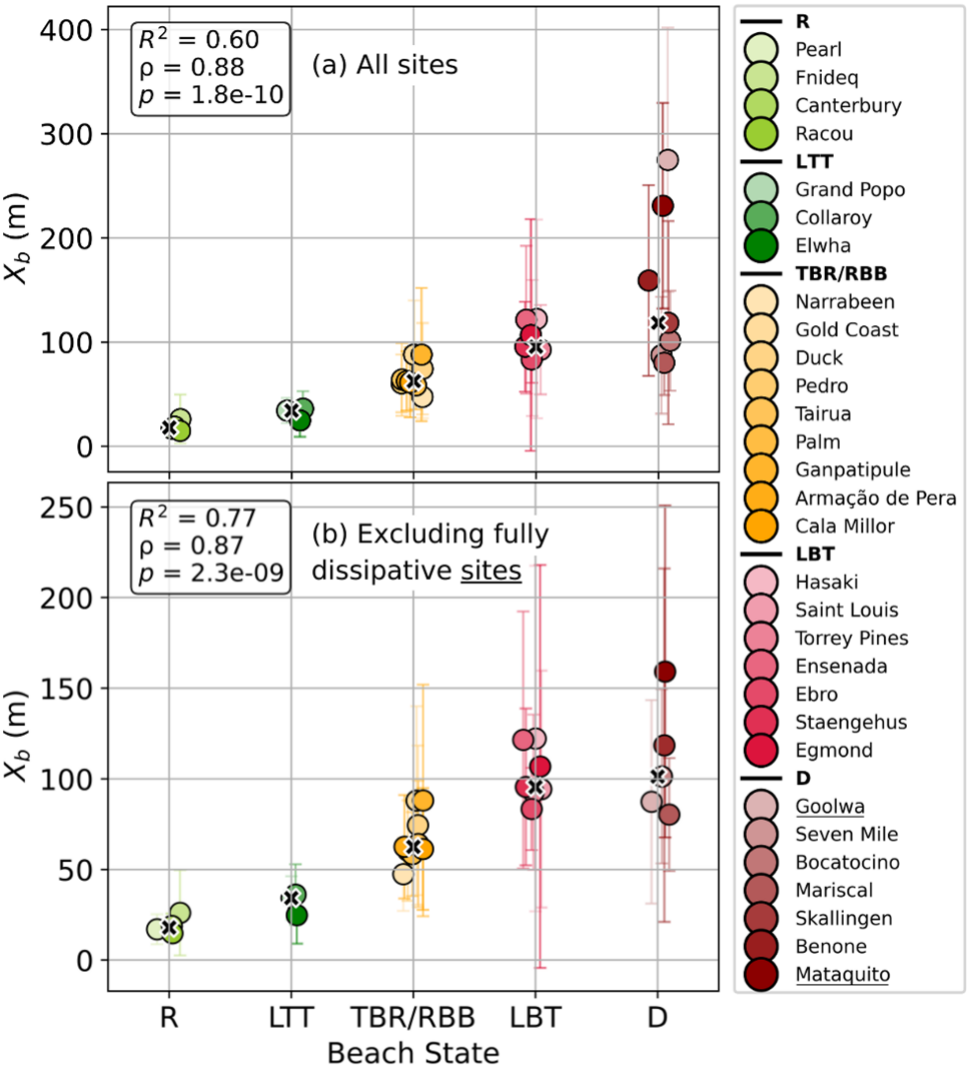
Relationship between median beach states from the literature and median satellite-derived cross-shore distance $X_b$. Circular markers represent individual validation sites, colored according to their known classified beach state: light green for R, dark green for LTT, orange for TBR/RBB, crimson for LBT, and dark red for D. Within each beach state class, color shades vary to differentiate individual sites. The black crosses indicate the median $X_b$ value for each class. Error bars denote the temporal standard deviation of $X_b$ across the full satellite time series for each site.

The cross-shore distance between the shoreline and the offshore breaking point ($X_b$) is examined as a proxy for morphodynamic state classification. $X_b$ aids to capture both wave-driven hydrodynamic forcing (via the breaking position) and nearshore morphological setting (through its cross-shore extent). Figure 2**(a) **shows median $X_b$ values from the time series of satellite observations at the 30 validation sites, plotted against their known most occurent beach states from the literature (column ’Beach state’ in Table 1). A correlation between median $X_b$ values and the corresponding median beach state from the literature resulted in a coefficient of determination of $R^2 = 0.60$ (where beach states from literature were assigned ordinal values: R = 1, LTT = 2, TBR/RBB = 3, LBT = 4, D = 5). Although this method assumes equal spacing between states, the high Spearman’s rank correlation coefficient ($\rho = 0.88$, $p = 1.8 \times 10^{-10}$) shows a monotonic relationship, showing that $X_b$ increases with greater dissipativeness. Beyond median values, Figure 2 also displays temporal standard deviations of $X_b$ for each site as vertical bars and shows that reflective beaches have limited temporal variability and increases gradually as beaches become more dissipative. This pattern reflects the dynamic nature of energetic beaches, which often shift between reflective, intermediate and dissipative states over time. In addition, Figure 2**(b)** presents the same validation after excluding two extreme cases (ultra/highly dissipative): Goolwa (Australia) and Mataquito (Chile). The dissipative conditions at these sites, characterized by wide surf zones and large $X_b$ values^66,68^, remain largely stable over time, unlike other dissipative beaches in the literature (e.g., Bocatocino, Seven Mile, and Skallingen) that exhibit temporal variability and shift between states. Consequently, their higher median $X_b$ values reflect the persistent nature of these sites, in contrast to other dissipative beaches that vary between states. With this new relationship, better determination coefficient ($R^2 = 0.77$) is found and the monotonic trend remains strong ($\rho = 0.87$, $p = 2.3 \times 10^{-9}$). Basically, $X_b$ reflects whether the bar is morphodynamically active, i.e., whether waves break on it or not. Under low-energy conditions, a bar may exist but remain inactive, yielding a small $X_b$ (shore breaking) and classifying the beach as reflective, even if the underlying morphology itself is intermediate. Rather than a limitation, this introduces a new perspective: $X_b$ captures the *active* beach state as controlled by instantaneous wave-bar interactions (discussed in Section Limitations and Perspectives).

**Fig. 3:**
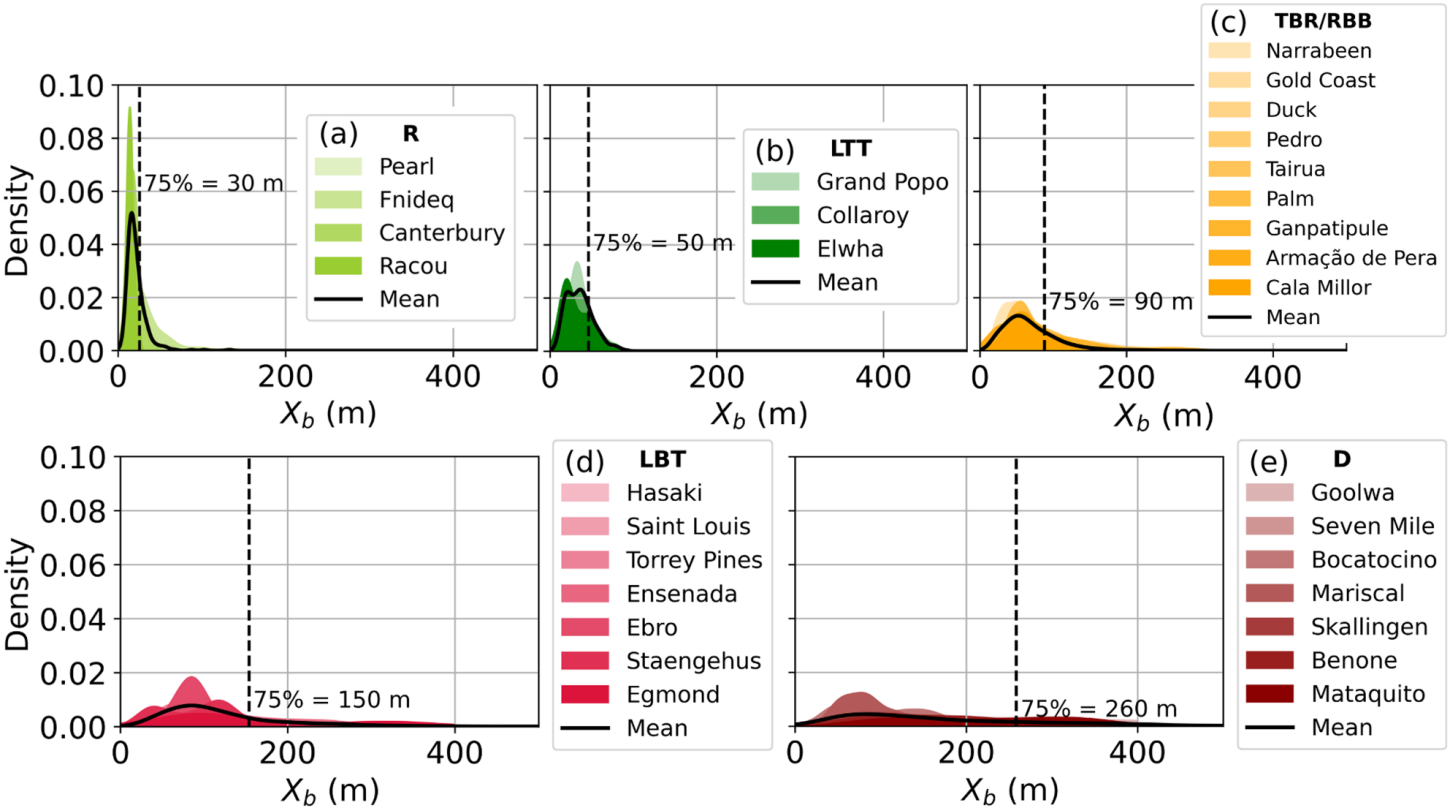
Time series distribution of $X_b$ per site and grouped by beach state (**(a)** R, **(b)** LTT, **(c)** TBR/RBB, **(d)** LBT and **(e)** D). The black line shows the median across site distributions per state and the dashed line indicates the 75th percentile of the median distribution.

The continuous proxy $X_b$ is converted into discrete morphodynamic classes using percentile-based threshold values representative of each beach state. To derive these thresholds, sites were first grouped according to their most recurrent (median) beach state reported in the literature. For each site, the full $X_b$ time series was used to construct a kernel density estimate (KDE), approximating its probability density function (PDF). Within each morphodynamic class, the site-specific PDFs were then averaged to obtain a single representative distribution per state, giving equal weight to each site. Thresholds were finally defined from the 75th percentile of these unique state distributions. This approach ensures that thresholds reflect the overall distributional behaviour of $X_b$ within each morphodynamic state, rather than relying solely on median values. The shape of the $X_b$ distributions differs across beach states. Reflective beaches where waves break near the shoreline show narrow distributions (Figure 3**(a)**). Moving toward more dissipative states, the distributions progressively widen and flatten, reflecting broader surf zones and the presence of bar systems (Figure 3**(b)**,**(c)**,**(d)**). Dissipative beaches display particularly broad distributions, indicative of high morphological variability through time (Figure 3**(e)**). The 75th percentile is selected as a balanced with both observations reported in the literature and the spatial extent of wave breaking typically observed on satellite imagery. The robustness of this choice is evaluated in Figure S8 in Supplementary material, which shows how threshold values vary with the selected percentile. Thresholds remain quite stable across the 70-80th percentile range: the transition from R to LTT occurs around 20–30 m, from LTT to TBR/RBB $\sim$50 m, from TBR/RBB to LBT between 80–100 m, and from LBT to D between 140–170 m. The narrow distributions associated with R, LTT and TBR/RBB states yield consistent thresholds, whereas the broader distributions of LBT and D states naturally lead to higher variability in percentile-based thresholds. The following approximate $X_b$ thresholds (in meters) were identified: $0< X_{b,R}< 30< X_{b,LTT}< 50< X_{b,TBR/RBB}< 90< X_{b,LBT}< 150< X_{b,D} < +\infty$. The threshold for the dissipative state ($X_{b,D} < 260$ m) does not mean anything physically because waves can break at any offshore distance, so there is no clear cut limit.

### Beach state variability and stability

**Fig. 4:**
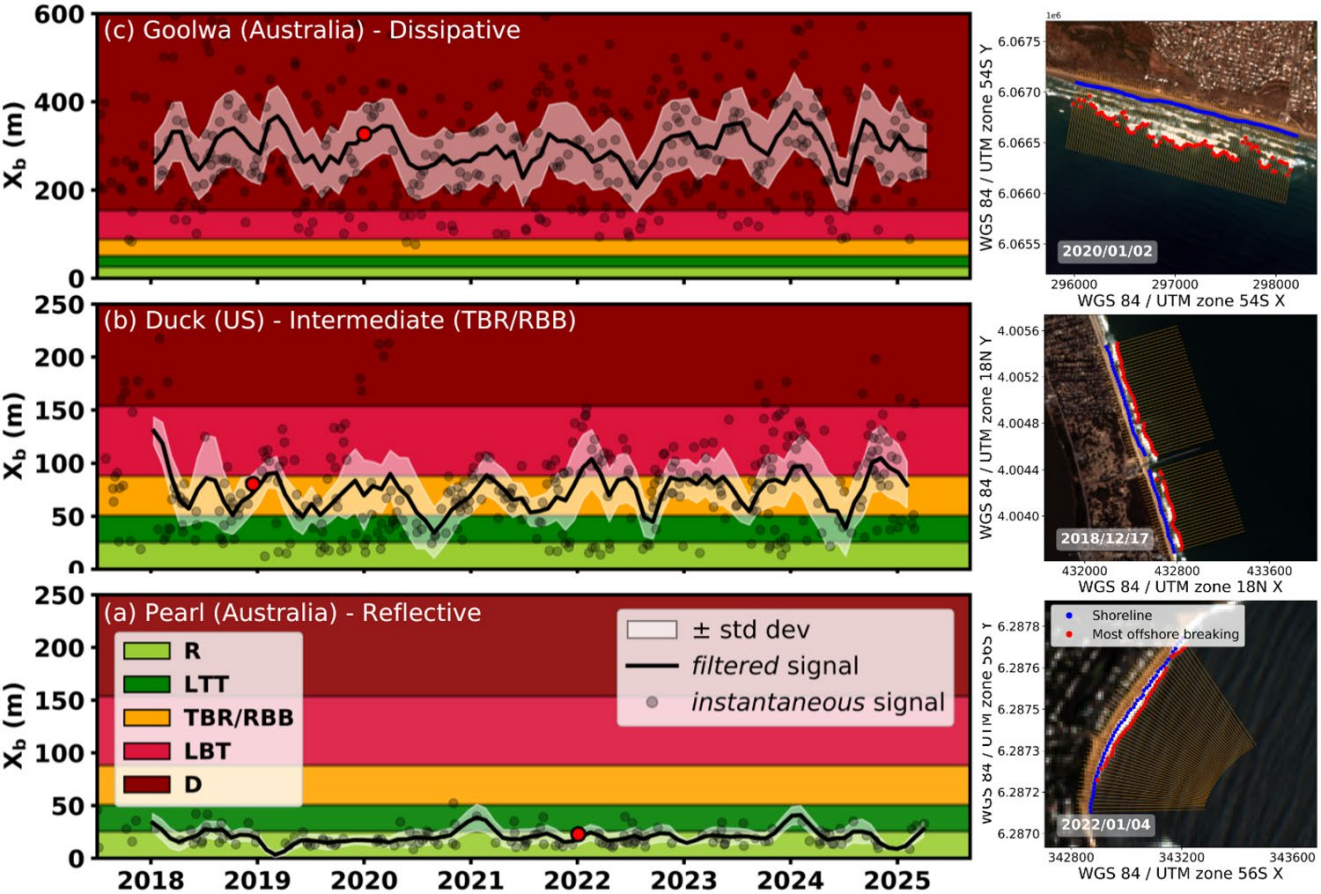
Time series of $X_b$ across the three representative sites: **(a)** Pearl in Australia, **(b)** Duck in North Carolina in the US and **(c)** Goolwa in Australia. The time series start in 2018 because between 2015 and 2018, only Sentinel-2A was available, providing very few images; this limited sampling would not allow for a consistent calculation of the 3-month moving average (*filtered* signal).

To illustrate the temporal variability of the $X_b$ proxy and its ability to capture beach state transitions, three representative examples are shown: Pearl (Australia), Duck (US), and Goolwa (Australia), classified in the literature as reflective, intermediate (TBR/RBB), and dissipative, respectively. For each site, the $X_b$ time series is shown in Figure 4 using black dots (raw data), along with a 3-month moving average (black line) and its associated standard deviation (white shading). In the background, coloured bands represent the morphodynamic beach states assigned to each $X_b$ value based on the thresholds defined earlier. A representative satellite detection is shown for each site, highlighting the shoreline and the offshore breaking detections. The distance between them defines the $X_b$ (shown as a red dot on the time series), which is the median across all transects. The raw $X_b$ time series (black dots) reveal strong variability, with sometimes abrupt transitions between reflective and more dissipative beach states. As previously mentioned, this variability reflects the instantaneous activity of the beach system rather than actual morphological change, depending on whether the sandbar is active, i.e. it interacts with incoming wave energy (e.g., through wave breaking over the bar). For example, in double-bar systems, the outer bar may be present but remain inactive under low wave energy, with dissipation occurring instead on the inner bar or directly at the shoreline (shore breaking). Similarly, even in single-bar systems, breaking might not occur on it, with breaking that may occur only on the shoreline. In both situations, $X_b$ decreases, leading to a more reflective profile despite an intermediate underlying morphology. Under more energetic conditions, breaking shifts seaward as the bar system becomes active again, producing abrupt increases in $X_b$. This mechanism explains the sharp fluctuations observed in the raw signal, particularly at multi-bar sites such as Duck and Goolwa. This justifies our use of the term *active* beach state, to emphasize that $X_b$ captures the functional beach state (the actual location of breaking), rather than just its static submerged morphology. Accordingly, a 3-month moving average is applied to the $X_b$ time series, filtering out high frequency sub-seasonal variability while emphasizing longer-term trends in bar/state activity. This filtering does not remove the information related to beach activity. The analysis distinguishes two complementary signals (see Section Methods [6]): the *instantaneous signal* (raw $X_b$, black dots) and the *filtered signal* (3-month moving average $X_b$, black line).

Pearl Beach, an unbarred pocket beach located within the Broken Bay estuary (NSW, Australia) is considered to have a predominantly reflective modal state that it maintains year round^85^. Field observations by Aagaard et al. (2013)^48^ reported a dimensionless fall velocity $\Omega = 1.7 \pm 0.8$, which corresponds to R-LTT transition. Figure 4**(a)** matches this description, with states oscillating between R and LTT ($X_b < 50 m$) over the entire Sentinel-2 observation period. Episodic LTT state changes are in fact observed (e.g., 2021, 2024), consistent with the findings of Aagaard et al. (2013)^48^ in 2011, who described that storm events has the potential to erode the beach face and drive the deposition of sediment within the shallow nearshore creating a terrace. These transitions suggest that even morphologically stable reflective systems may temporarily reorganize under energetic forcing. Goolwa Beach, oriented toward the southwest, directly facing the dominant swell from the Southern Ocean, features a highly energetic and extremely dissipative beach. While it remains consistently dissipative in state, it exhibits substantial temporal variability in surf zone dynamics. The surf zone spans 400 m in width, with spilling breakers often observed over two longshore continuous bars with persistently high $X_b$ values (> 200 m). Figure 4**(c)** illustrates this other end-member case: while some shifts into LBT are visible in the raw data, the beach is generally classified as dissipative year round^66^. In contrast to Pearl and Goolwa, which exhibit relatively stable state time series, Duck Beach is known for its high temporal variability in terms of beach state. The site typically features a dynamic inner bar and, at times, a more stable outer one. This outer bar may decay via NOM following storm events or high-energy wave regimes. Figure 4**(b)** is consistent with this temporal variability, showing frequent transitions between R, LTT, TBR/RBB, and LBT with the 3-month moving average with ±1 standard deviation envelope $X_b$ ranging from $\sim$10 to 150 m. This is in line with prior observations by Lippmann and Holman (1990)^21^. The *instantaneous signal* of $X_b$ captures the two known NOM at Duck, in 2018 and 2020^86^, each corresponding to the formation of a new sandbar under energetic wave conditions. The use of a moving average proves particularly effective here, as it smooths over the NOM events themselves, focusing instead on the broader, more stable beach-state transitions, rather than the rapid dynamics of individual bar migrations.

For each site, the probability of occurrence of each beach state $P_s$ can be estimated as the ratio between the number of days where beach state *s* is observed and the total number of available days. By introducing $N_s$, the number of transitions into a different state, and $\tau _s$ the average residence time in state *s*, $P_s$ can also be expressed as:

12 $$\begin{aligned} P_s = \frac{N_s \langle \tau _s \rangle }{\displaystyle \sum _{k} N_k \langle \tau _k \rangle } \end{aligned}$$

**Fig. 5:**
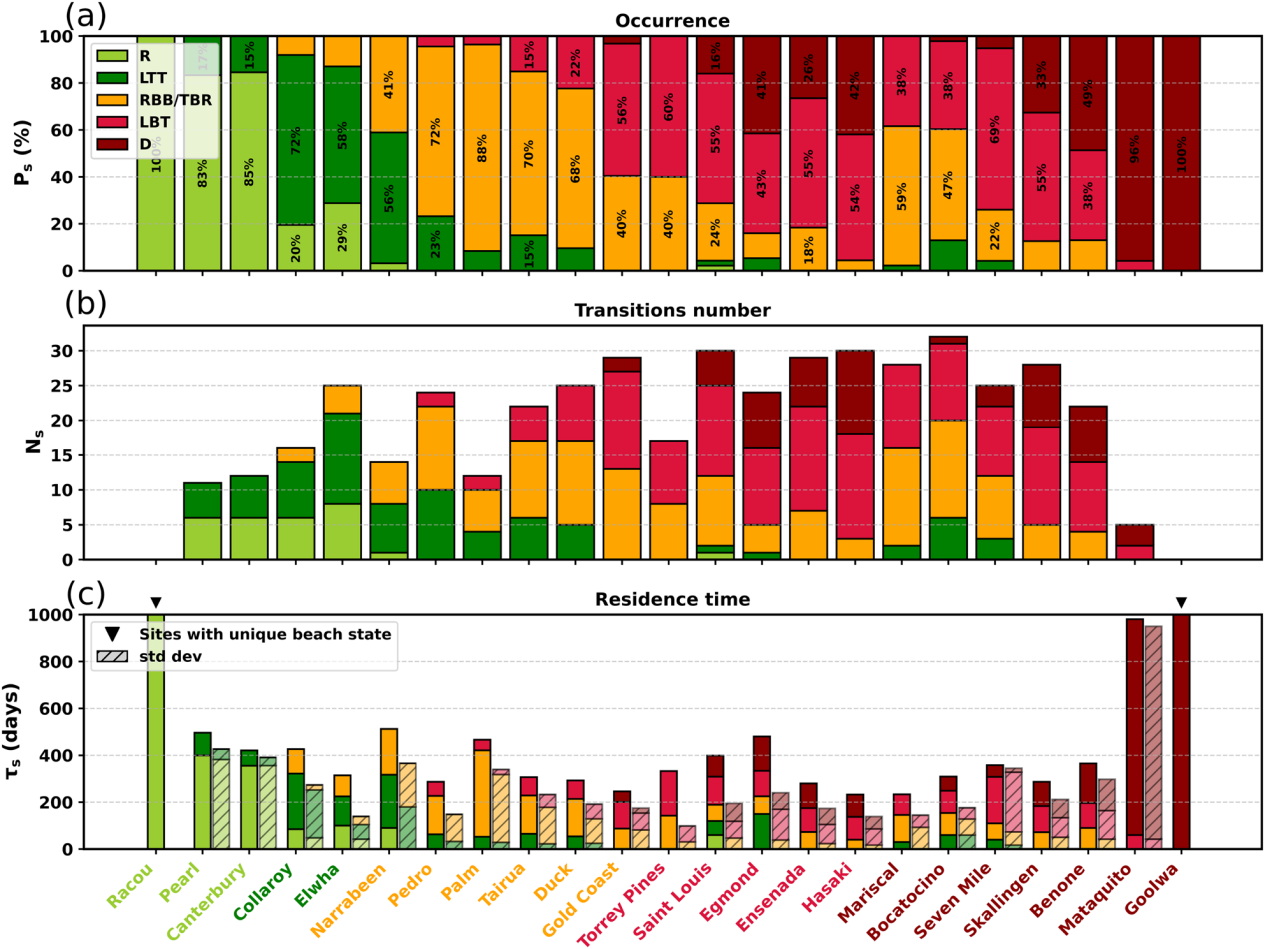
Bar charts presenting three key metrics: **(a)** occurrence probability of each beach state ($\%$), **(b)** number of state transitions, and **(c)** mean residence time (days). Only states with occurrence probabilities above 15$\%$ are shown. Black triangles show residence times greater than 1000 days that are truncated. Solid bars indicate the mean residence time, while hatched bars represent the standard deviation.

Figure 5 shows these three metrics. The *filtered signal* of $X_b$ (3-month moving average) was used to compute the different terms of Eq. 12. In addition, Table **S1** in Supplementary material lists all study sites, sorted by region and latitude, and reports the percentage of images in which wave breaking has been detected. For these calculations, only sites with more than 20$\%$ of images showing breaking (see Limitations and Perspectives for details), except for Racou, which was included as a representative fully reflective end-member despite its low percentage of detectable breaking. Racou is in fact reflective due to its sheltered setting and limited wave exposure, resulting in infrequent observable breaking in satellite imagery. The extreme sites that exhibit a unique beach state: the fully reflective Racou site in southern France and the fully dissipative Goolwa site in Australia are marked with small black triangles (Figure 5**(c)**). Their residence time corresponds to the total period covered by Sentinel-2 observations (i.e. 3425 days for Racou and 3401 days for Goolwa) and consequently their number of transitions is necessarily zero. Mataquito, also considered as a highly dissipative, with few observed LBT states, exhibits a sharp increase in residence time. Except those sites, the residence time of the more dissipative states (LBT and D) appears relatively short, whereas the more reflective states (R, LTT, and TBR/RBB) tend to persist for longer periods (Figure 5**(c)**). Often, the nearshore morphology is reset by high-energy wave regime leading to an LBT or D state regardless of the previous beach state. Once wave energy decreases, the shore-parallel sandbar develops rhythmic structures and the morphology transitions from an LBT state toward a TBR/RBB state. Under sustained low-energy wave conditions, the morphology then evolves toward an LTT or even R state. However, the occurrence of a strong wave regime at any stage of this cycle could immediately reset the morphology back to an LBT or D state. This explains the short residence times observed, which can be interpreted as a measure of linear bar persistence or a form of morphological stability^26^. The large standard deviations associated with the estimated mean residence times (indicated by the hatched bars) show that residence times are highly variable across all beach states and sites. This variability is consistent with the findings of Lippmann and Holman (1990)^21^ and Ranasinghe et al. (2004)^26^. Sites characterized as more dissipative (dominated by LBT and D states) like Saint-Louis, Bocatocino or Hasaki exhibit a higher total number of transitions (Figure 5**(b)**). This suggests that these beaches are morphodynamically less stable, with more frequent state changes. In contrast, steeper beaches (R, LTT) like Collaroy or Canterbury show fewer transitions, pointing to greater stability. Transitions are generally concentrated in the intermediate states (LTT, TBR/RBB, LBT) which indicate morphodynamically unstable configurations that beaches frequently pass through when oscillating between the more stable reflective and dissipative extremes.

Similar studies have been conducted using video systems on several beaches in Australia (Collaroy, Narrabeen, Seven Mile, Goolwa^11^, Palm Beach^26^), as well as at Duck^21^. These previous results are compiled in Table 2, together with the present occurence estimates (column ’Here’). In the referenced studies, TBR and RBB states were reported separately; their occurrences were therefore combined to match the classification scheme adopted here. In the case of Lippmann and Holman (1990)^21^, the LBT and TBR states defined by Wright and Short (1984)^11^ were further subdivided into bar types G (infragravity scaled 2D bar) and F (non-rythmic 3D bar) and types D (attached rythmic bar) and C (non-rythmic attached bar), respectively. For consistency, these sites were merged into a single LBT state (F, G) and a combined TBR/RBB state (C, D, E (offshore rythmic bar)). Overall, the values found for the occurrence probability ($P_s$) are generally in good agreement with those reported in the literature (Table 2). Near-zero probabilities are also observed for states not reported at Duck, Palm Beach, or Collaroy. The largest discrepancy appears at Narrabeen where Wright and Short (1984)^11^ report $P_{TBR/RBB} = 70\%$ compared to 41$\%$ in our results.

**Table 2:** Beach state occurrences at Duck, Palm, Collaroy, Narrabeen, Seven Mile and Goolwa.

Occurence	Duck		Palm		Collaroy		Narrabeen		Seven Mile		Goolwa	
($\%$)	Ref^21^.	Here	Ref^26^.	Here	Ref^11^.	Here	Ref^11^.	Here	Ref^11^.	Here	Ref^11^.	Here
R	0	0	0	0	20	20	5	3	0	0	0	0
LTT	9	10	5	8	50	72	20	56	0	4	0	0
TBR/RBB	70	68	83	88	30	8	70	41	40	22	0	0
LBT	21	22	12	4	0	0	3	0	50	69	10	0
D	0	0	0	0	0	0	2	0	10	5	90	100

**Table 3:** Residence times of beach states at Duck and Palm.

Residence time	Duck		Palm	
(days)	Ref^21^.	Here	Ref^26^.	Here
R	0	0	0	0
LTT	9,3	54	18	52,5
TBR/RBB	34,6	160	12,1	368,5
LBT	5,4	78,75	2,5	45
D	0	0	0	0

Although the proportions of beach states observed at Palm Beach and Duck are consistent with those reported in the literature, the mean residence times ($\tau _s$) derived from satellite data are significantly higher (for example, at Duck $\tau _{LTT} = 54$ days compared to 9.3 days^21^, Table 3). This discrepancy reflects the use of a three-month moving average to compute residence times, which filters out short-term variability and emphasizes persistent morphological states. Unlike ARGUS video systems, which provide high-frequency monitoring (up to every 10 minutes) and can directly capture instantaneous beach state transitions, Sentinel-2 imagery offers a much lower and irregular temporal resolution (theoretically 5 days, but often ranging from a few days to over a month due to te available images: cloud-free and visible breaking waves, see Section Methods [7]). As a result, the *instantaneous signal* of $X_b$ signal is too intermittent to derive meaningful residence times, making temporal smoothing necessary to capture sustained phases of beach activity. Consequently, $\tau _s$ derived from our approach reflect lower-frequency morphological stability rather than the full spectrum of short-term dynamics accessible from daily monitoring. This highlights both the limitations and the complementarity of satellite-based approaches in characterizing coastal morphodynamic behavior.

Validation against the CNN-based classifications of Ellenson et al. (2020)^27^ showed that the satellite-derived $X_b$ successfully reproduces the seasonal variability of beach states, with Mean Absolute Errors (MAE) of 8$\%$ (R = 0.76) at Duck (Figure **S2(a)**) and 11$\%$ (R = 0.66) at Narrabeen (Figure **S2(b)**). These errors, expressed as percentage differences in the monthly proportion of each beach state, indicate a generally good agreement with the CNN reference. A more detailed explanation is provided in the Supplementary material.

### Morpho-sedimentary parameters

**Fig. 6:**
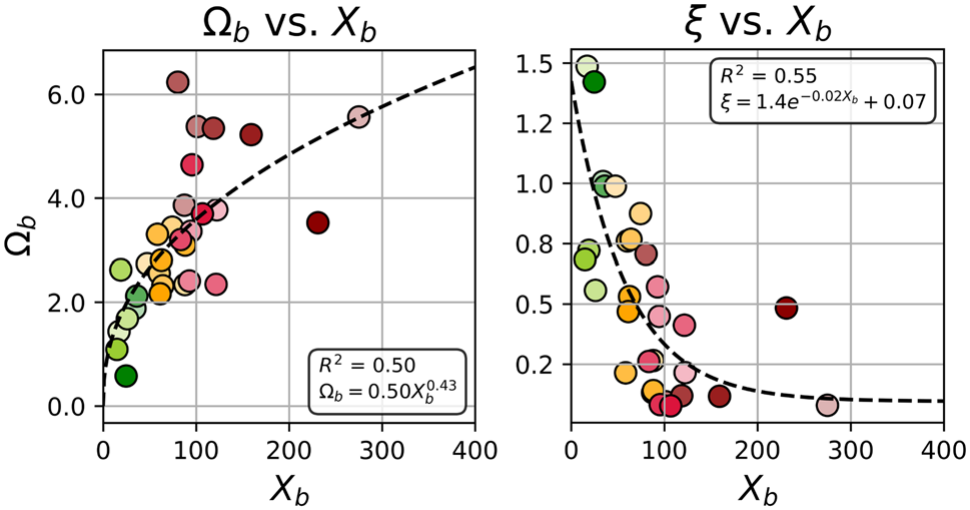
Empirical relationships between satellite-derived beach state proxy $X_b$ and classical dimensionless parameters: (**a**) sediment fall velocity parameter $\Omega _b$ and (**b**) Iribarren number $\xi$.

Beach states classify morphodynamic configurations related to their hydrodynamic setting, providing qualitative insight into coastal setting and characteristics. Inversely, one can use a beach state classification (e.g. the beach state proxy $X_b$) to get a qualitative idea of the local morpho-sedimentary parameters. For example, estimating essential morphological parameters at sites lacking in-situ measurements: the sediment median grain size ($D_{50}$) and the beach slope ($\tan \beta$). While wave parameters ($H_s$, $T_p$) are available from global reanalysis wave hind-cast products such as ERA5^78^ or CMEMS^87^, sediment grain size and beach slope are typically site-specific and rarely measured across the world. To address this gap, two empirical relationships are proposed, linking these morphological parameters to $X_b$ in combination with hydrodynamic forcing. A review was conducted on 30 well-documented sites (Table 1), reporting median grain size ($D_{50}$) and beach slope ($\tan \beta$). The dimensionless fall velocity ($\Omega _b$) and the Iribarren number ($\xi$) were computed, and their dependence on $X_b$ was assessed. As illustrated in Figure 6, the analysis revealed two empirical relationships: the dimensionless fall velocity $\Omega _b$ scales with $X_b$ following a power law $\Omega _b = a_1 X_b^{a_2}$, whereas the Iribarren number $\xi$ is well described by a decay exponential function $\xi = c_1 e^{-c_2 X_b} + c_3$. These relationships allow inverting the problem to express both sediment grain size ($D_{50}$) and beach slope ($\tan \beta$) as functions of $X_b$, $H_s$, and $T_p$ only. The methodology is detailed in Methods [8] and the choice of these empirical models is discussed in section Limitations and Perspectives.

**Table 4:** Summary statistics (P25, median, P75) of $H_s$, $T_p$, $D_{50}$, and $\tan \beta$ by beach state.

	$H_s$ (m)			$T_p$ (s)			$D_{50}$ ($\mu$m)			tan$\beta$		
	P25	Median	P75	P25	Median	P75	P25	Median	P75	P25	Median	P75
R	0.66	1.07	1.37	6.1	7.5	8.3	560	710	885	0.10	0.12	0.13
LTT	0.75	1.05	1.35	6.2	7.3	8.5	305	380	465	0.07	0.08	0.09
TBR/RBB	0.85	1.09	1.41	6.6	7.7	9.0	180	230	280	0.04	0.05	0.06
LBT	0.85	1.12	1.45	6.6	7.9	9.5	110	140	175	0.018	0.02	0.03
D	1.16	1.45	1.87	7.6	8.9	10.1	60	80	100	0.008	0.010	0.012

The *instantaneous signal* of $X_b$ (raw data) is used without any moving average to capture the instantaneous variability of beach state and associated forcing conditions, enabling the reconstruction of time series for grain size and beach slope using Eqs. 10 and 11. After retrieving time series of $H_s$, $T_p$, $D_{50}$, and $\tan \beta$, their cross site distributions were calculated for all beach states across the 30 sites. For every state (R, LTT, TBR/RBB, LBT, and D), all respective data from all sites were aggregated, and the 25th percentile (P25), median, and 75th percentile (P75) were computed. Table 4 illustrates these typical value ranges, offering a concise reference for the hydro-sedimentary characteristics of each state. Median significant wave height ($H_s$) ranges from $\sim$1.1m (R) to $\sim$1.5m (D) across the five beach states with intermediate beach states (LTT, TBR/RBB, LBT) showing increasing $H_s$ from reflective to dissipative conditions. Peak period ($T_p$) also exhibits the same pattern, rising from $\sim$7.5s (R) to $\sim$8.9s (D). Median grain size ($D_{50}$) decreases from $\sim$700$\mu$m for R to $\sim$80$\mu$m for D, and median beach slope ($\tan \beta$) decreases from $\sim$0.12 to $\sim$0.01. Intermediate beach states (LTT, TBR/RBB, LBT) show progressively decreasing $D_{50}$ and beach slope from reflective to dissipative conditions. These median values are broadly consistent with ranges reported in the literature, though sediment sizes for dissipative states remain relatively low, likely reflecting limitations of the empirical model used (discussed in Limitations and perspectives). A seasonal-cycle validation of the reconstructed beach slope is provided for four reference sites (Torrey Pines, Duck, Narrabeen, and Collaroy) in Figure** S5** in the Supplementary material, showing encouraging agreement at several sites.

**Fig. 7:**
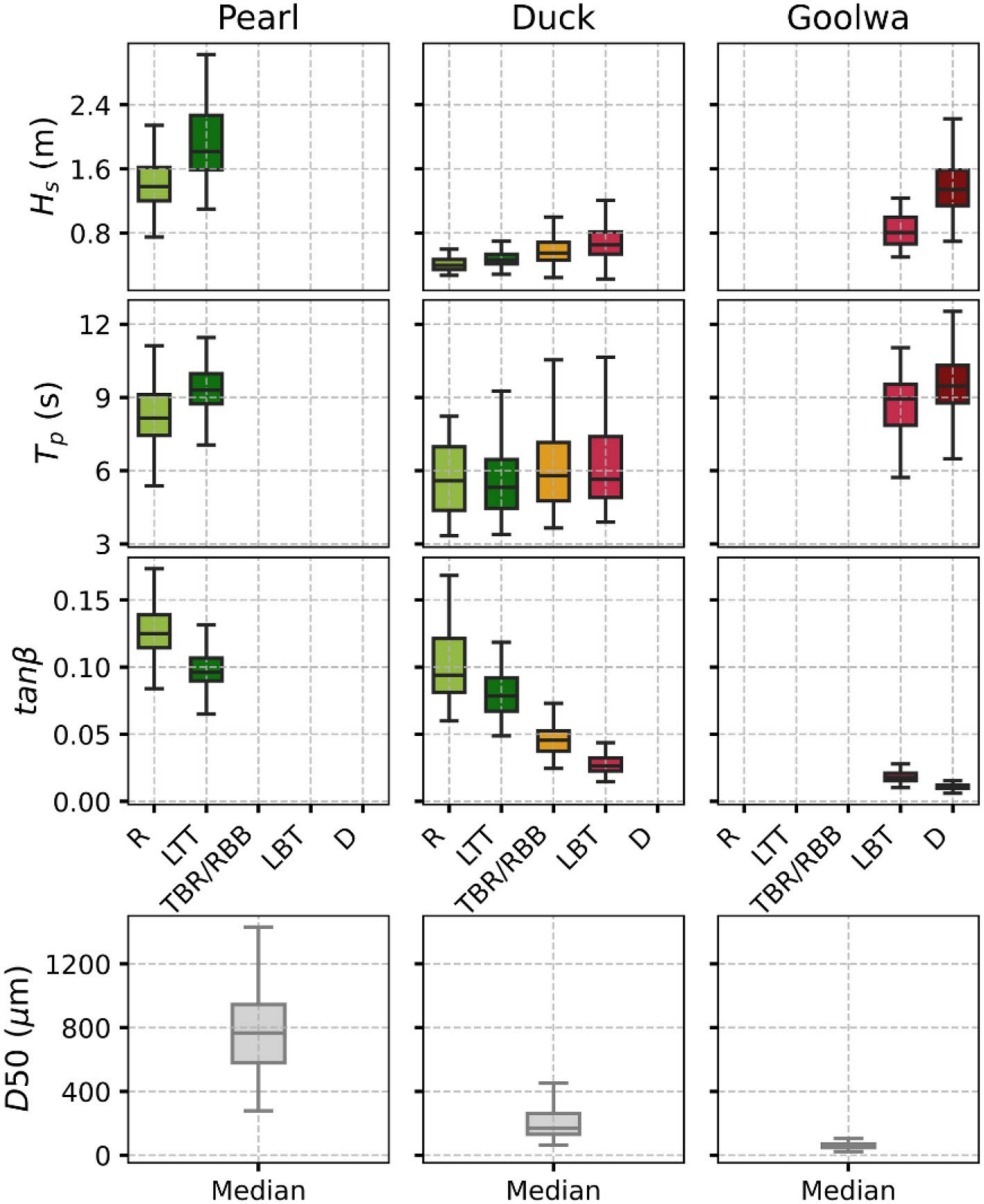
Boxplot distributions of wave and morpho-sedimentary parameters across beach states for the three representative sites: Pearl, Duck and Goolwa.

To complement the cross-site analysis, site-specific variability in hydro-morphodynamic parameters is illustrated for Pearl, Duck, and Goolwa. In Figure 7, each column corresponds to one site, and each row represents one of the four hydro-morphodynamic variables ($H_s$, $T_p$, $D_{50}$ and $\tan \beta$). Within each subplot, box-plots show the distribution of that variable across the different beach states observed at that site, i.e. the number of box-plots for each subplot varies with the number of states the site experienced. Pearl, for example, experienced only two states (R and LTT) like Goolwa (LBT and D) while Duck shows four beach states (R, LTT, TBR/RBB and LBT). Only states observed at least 25 times were included, ensuring consistent computation of the box-plot. For $D_{50}$, only one box-plot per site is shown, since the empirical model tends to exaggerate state-dependent variability in sediment size; instead, the site median is retained as a more realistic indicator (discussed in the Section Limitations and Perspectives). For $H_s$, as expected, the three sites show increasing wave height as the beach becomes more dissipative. The IQR also increases, indicating stronger variability in wave forcing. At Duck, the results are consistent with Lippmann and Holman (1990)^21^: linear bars are associated with the highest waves, bars with longshore variability with intermediate energy, and the reflective state with the lowest waves. At Pearl, $H_s$ ranges between 1.25 and 2.3 m, higher than the values observed at Duck or Goolwa. This likely reflects a known limitation of ERA5^78^, which does not properly resolve wave conditions in sheltered coastal environments like Pearl (discussed in Limitations and perspectives). Regarding beach slope, despite the limitations of ERA5^78^, Pearl is relatively well captured, with values >0.1 for the reflective state and $\sim$0.1 for the LTT state^48,88^. At Duck, four distinct slopes are observed: $\sim$0.09 for R, $\sim$0.08 for LTT, $\sim$0.05 for TBR/RBB, and $\sim$0.03 for LBT. Finally, at Goolwa, slopes are very low, 0.017 for LBT and 0.01 for D, consistent with its highly dissipative nature^66,67^. The large spread of $D_{50}$ values for reflective site (Pearl) versus the narrow range for strongly dissipative site (Goolwa) is consistent with morphodynamic expectations: reflective beaches often contain coarser and more heterogeneous material, whereas dissipative beaches are typically finer and more homogeneous^88^. However, the empirical inversion applied here may amplify state-dependent variability in $D_{50}$; these patterns should be interpreted with caution. In fact, for sediment grain size ($D_{50}$), the model reproduces general trends across the three representative sites but experiences some discrepancies with literature. For Pearl Beach, the model predicts a median $D_{50}$ of $\sim$760$\mu$m, higher than the in-situ value of 300$\mu$m. At Duck, the simulated $D_{50}$ ($\sim$170$\mu$m) is close to the observed 150$\mu$m. At Goolwa, the model predicts $\sim$60$\mu$m, less than the in-situ measurement of 120$\mu$m. These inaccuracies are due to the empirical formulation used, increasing contrasts between beach states, but overall the model maintains the relative size hierarchy of sediment from reflective to dissipative beaches.

## Limitations and perspectives

Deducting $X_b$, by combining wave breaking morphology and its location, enables the first order detection of the *active* state of the beach. The beach states defined in this study highlight distinct configurations of the beach system and encompass the full range of known morphological settings, from fully dissipative to fully reflective conditions (as proposed by the models^11,21^). However, the current approach misses the resolution to separate beach states into sub-states like TBR and RBB. In practice, the spatial resolution and instantaneous nature of satellite-derived breaking patterns make such discrimination challenging (see Figure **S7** and detailed explanation in the Supplementary material). This is mainly due to the inability to detect the 3D-ness of the bars, only based on instantaneous breaking.

Figure 2 shows that $X_b$ broadly reproduces the locally grounded beach states by former studies. However, it is important to note the partly subjective nature of the “median state” as reported by different authors. For some sites, where dedicated studies have been carried out to characterize beach state, the median state is well established and can be assigned with confidence. For others, it depends more heavily on expert judgment and may vary depending on the observer or the time period considered. This heterogeneity in reference sources must be kept in mind when interpreting both the agreements and the occasional discrepancies between $X_b$ and the literature. Some sites (e.g., Goolwa, Mataquito) remain in a single beach state throughout the time series, in line with local knowledge, while others reported as dissipative in the literature are observed to cycle through multiple states in our dataset.

The universal thresholds defined in Figure 3 ensure consistency across the diversity of the 30 study sites and support transferability to other regions. The selection of the 75th percentile provides an objective criterion, whose robustness is demonstrated in Figure S8 in the Supplementary Material. Threshold values were found to be stable whether unique state distributions were constructed by pooling all observations or by averaging site-specific densities, indicating limited sensitivity to the aggregation method. Nevertheless, alternative approaches (e.g., Bayesian inference or multi-criteria clustering) could refine threshold estimation in future work. Nevertheless, if the method is applied to a specific region, these thresholds can be adapted to this particular area to better reflect the local conditions.

The statistics shown in Figure 5, including beach-state occurrences, transition counts, and residence times, could directly inform how beaches may be monitored. Highly dynamic beaches may require frequent satellite acquisitions or, where possible, in-situ sensors to capture rapid changes, whereas stable beaches can be monitored less intensively. $X_b$ may help identify, at least as a first estimate, which monitoring strategy is the most appropriate. However, this approach is inherently limited by the availability of a sufficient number of images containing visible wave breaking, as the 3-month moving average must remain physically meaningful. To assess this limitation, Table** S1** in the Supplementary material presents, for each site, the percentage of cloud-free images and, among them, the proportion of images containing visible breaking. The table is organised by region, and within each region, sites are ordered by latitude. The proportion of cloud-free images generally exceeds 50$\%$ for sites located below 50° latitude. For NE sites such as Benone, Druridge Bay, and Egmond aan Zee, the reduced availability of clear images indicates that satellites may not be the most suitable tool for high-frequency monitoring. Also, in the MED region, the proportion of images with visible breaking remains particularly low, averaging less than 7$\%$. This greatly limits the applicability of the method in such environments, where the absence of breaking may itself carry physical meaning that could be further explored in future work. Overall, the relatively high breaking images percentages observed across most selected sites are encouraging, as they suggest that the method could be extended to larger spatial scales (see Section Methods [7]).

The potential of $X_b$ to provide first-order estimates of sediment grain size and beach slope was explored through empirical relationships linking $X_b$ to $\Omega _b$ and $\xi$. Among several candidate functional forms (linear, logarithmic, exponential, power law), a power law best described the relationship between $\Omega _b$ and $X_b$, while an exponential decay provided the most consistent fit for $\xi$. Physically, $\Omega _b$ increases with $X_b$, while $\xi$ decreases as $X_b$ increases, consistent with the transition from plunging to spilling breaker regimes across reflective to dissipative states. However, the exponential formulation predicts a maximum $\xi$ of approximately 1.5 as $X_b \rightarrow 0$, whereas classical theory associates strongly reflective conditions with $\xi> 3.3$^16,17,89^. This limitation reflects the constraints of the available dataset rather than the underlying physics and suggests that more complex formulations may be required to capture extreme reflective conditions. Additional uncertainty arises from the use of ERA5 wave forcing^78^, which may overestimate nearshore wave conditions in sheltered environments (e.g., Pearl). Because $H_s$ appears in Eq. 10 with an exponent of 4/5, such overestimation directly propagates into sediment-size estimates. Furthermore, $D_{50}$ depends on $\Omega _b$, which itself relies on the breaking wave height $H_b$ approximated using Komar’s (1974) empirical formulation^90^. These cascading approximations compound uncertainty in both $D_{50}$ and $\tan \beta$. Although $\xi$ depends directly on $H_s$ and is therefore not affected by uncertainties in $H_b$, it remains sensitive to the offshore forcing dataset. Recent studies in efficient nearshore wave propagation modelling, such as SnapWave^91^, offer the possibility to dynamically transform offshore wave conditions. Incorporating such approaches in future work would improve the representation of the effective wave forcing experienced at the shoreline, particularly in sheltered settings (e.g. Pearl, Racou).

In the Supplementary material, Figure **S5** shows the comparison of the seasonal cycles of beach face and nearshore slopes between in-situ profiles and satellite-derived $X_b$ estimates for four study sites (Torrey Pines, Collaroy, Narrabeen, and Duck). The satellite indicator generally captures much better the seasonal cycle of the beach face slope than the nearshore slope, with the strongest agreement at Torrey Pines ($R^2$=0.87) and moderate agreement at Collaroy ($R^2$=0.53), while correlations are weaker at Narrabeen and Duck but capturing the mean beach face slope. A more detailed analysis is provided in the Supplementary material.

While the present study focus primarily on wave-dominated beaches, it is essential to consider the role of tides in beach-state classification, especially if the goal is to assess beach states across larger scales^12^. To this end, the same methodology is applied to seven additional sites exhibiting meso- or macro-tidal ranges, encompassing tide-modified and tide-dominated beaches. Results show that the median $X_b$ increases with tidal range, reflecting broader surf zones and larger tidal excursions. Temporal variability, expressed as the standard deviation of $X_b$, also tends to increase with tidal range, indicating more dynamic and tide-dependent breaking patterns. These results highlight that interpreting $X_b$ becomes increasingly challenging in tide-modified environments, where wave breaking shifts with tidal stage. A detailed description of the sites, methods, and implications is provided in the Supplementary material, where Figure S9 illustrates this aspect. In addition, since $X_b=x_b-x_s$, uncertainties in shoreline position $x_s$, which increase at meso- and macro-tidal sites due to large tidal range, can further amplify the variability of $X_b$. Conversely, tidal corrections can also introduce uncertainties at microtidal sites where the tidal signal is small relative to other sources of shoreline variability^92^.

Our approach provides a first-order estimate of key morpho-sedimentary parameters, independently of in-situ data. The $X_b$ metric, by integrating sandbar and shoreline positions, could help improve shoreline evolution models by accounting the bar setting, information often neglected in traditional $\Omega _b$-based approaches (e.g^93,94^.,). Similarly, the estimated morpho-sedimentary parameters (grain size, beach slope) could provide initial and boundary conditions for numerical coastal models, such as CROCO^95^, particularly at data-poor sites.

Furthermore, an interesting extension of this work would be to compare $X_b$ with time-varying equilibrium indicators such as $\Omega _{eq}$^96^, which explicitly account for antecedent wave forcing and beach-memory effects. Sensitivity analyses on the characteristic memory timescale could help assess how hydrodynamic inheritance interacts with the morphologically active configuration captured by $X_b$, particularly if applied across large spatial scales.

Clearly, this method is valid only if waves are actively breaking over the bar, the terrace, or the shoreline. As discussed above, $X_b$ reflects the *active* beach state, i.e. how the morphology responds to the prevailing wave regime rather than the morphology itself. This idea raises a broader question: if a beach in the Mediterranean sea for instance retains a 3D bar system (TBR/RBB) for months under low wave energy, with no active circulation or rip processes, does it in practice behave more like a reflective beach? In this sense, the limitation of the method also opens a new way of considering what defines a beach state and its active state capturing the current interaction between waves and the underlying morphology.

## Conclusion

For this work we introduced the concept of *active* beach states, defined by the cross-shore distance from the shoreline to the offshore breaking wave position ($X_b$). This observable metric integrates wave forcing into beach state classification by capturing whether waves break on bars, terraces, or directly at the shoreline, thereby reflecting the functional response of the beach to prevailing hydrodynamics. Applied across 30 sandy wave-dominated microtidal beaches, $X_b$ demonstrates that beaches can be systematically classified from satellite imagery, following the frameworks of Wright and Short (1984), Lippmann and Holman (1990) and Ranasinghe et al. (2004) frameworks^11,21,26^. Using percentile-based thresholds, time series of beach states are derived, quantifying occurrences, residence times, and transition frequencies. Independent comparison with CNN-based classifications at Duck and Narrabeen^27^ further confirms that the seasonal variability of beach states is well reproduced.

Beyond classification, the *instantaneous signal* of $X_b$ provides first-order empirical estimates of beach slope ($\tan \beta$) and median sediment grain size ($D_{50}$). Although these estimates rely on simplified empirical formulations and require further validation, they offer morpho-sedimentary context at data-poor sites and may assist in model initialization or large-scale coastal assessments.

Overall, this study demonstrates that beach-state dynamics can be monitored from satellite imagery using a physically observable metric. By linking morphodynamic state to observable surf-zone processes, $X_b$ bridges the gap between static geomorphological classifications and dynamic wave-sediment interactions. While further refinement is needed, particularly regarding threshold optimization, empirical inversion, and application to tide-influenced systems, this framework provides a transferable foundation for regional to global monitoring of sandy coasts.

## Supplementary Information

Supplementary Information.

## Data Availability

The datasets used and analysed during the current study are available from the corresponding author on request.
