# Supplementary material for: Multi-site assessment of microtidal wave-dominated active beach state and morpho-sedimentary parameters using optical satellite imagery
Source: Sci Rep. 2026 Mar 27;16:10949. doi: 10.1038/s41598-026-45638-3 (PMC13039959; doi:10.1038/s41598-026-45638-3)
Supplement: Supplementary file 1 — Supplementary Information. [file 41598_2026_45638_MOESM1_ESM.pdf]

Multi-site assessment of micro-tidal wave-dominated active beach state and morpho-sedimentary parameters using optical satellite imagery

Salomé Frugier[1,*], Rafael Almar[1], Erwin W.J. Bergsma[2], Marcan Graffin[1,2], and Gerben Ruessink[3]

[1]LEGOS (Laboratoire d'Etudes en Géophysique et Océanographie Spatiales), Toulouse, France
[2]CNES (Centre National d'Etudes Spatiales), Toulouse, France
[3]Department of Physical Geography, Faculty of Geosciences, Utrecht University, Utrecht, The Netherlands
[*]Correspondence should be addressed to Salomé FRUGIER at salome.frugier@ird.fr.

ABSTRACT

Traditionally, beach states are defined from visual observations, in-situ measurements and/or video imagery, which limits their application to a handful of well-instrumented sites. In this work, we propose a different approach by focusing on a remotely observable quantity: the cross-shore distance between the offshore wave-breaking and the shoreline position, denoted X_b. This metric defines the *active* beach state, capturing where waves dissipate energy relative to the underlying morphology. Using 10 years of Sentinel-2 imagery, X_b is evaluated across 30 wave-dominated micro-tidal sandy beaches spanning reflective to fully dissipative conditions. The metric reproduces the structure of classical beach state frameworks and enables classification into five active states (R, LTT, TBR/RBB, LBT, and D) using transferable thresholds. X_b is continuous, thus it also reveals how beach state evolve through time, allowing quantification of state occurrence, residence time, and transitions, with seasonal variability consistent with independent classifications at well-studied sites. Furthermore, using empirical relationships, we demonstrate that X_b carries first-order information about beach-face slope ($\tan\beta$) and sediment grain size (D_{50}), opening a pathway toward systematic satellite-based monitoring of coastal morphodynamics at regional to global scales.

SUPPLEMENTARY SUMMARY

This supplementary document provides additional material supporting the methodology and results presented in the main paper. The content is organized as follows:

- **Figure S1** – Example of an image filtering based on cloud coverage at the FRF site, modified after[1].
- **Figure S2** – Seasonal cycles of beach state distributions at **(a)** Duck and **(b)** Narrabeen, comparing satellite-derived X_b classifications (Sentinel-2) with CNN-based classifications from Ellenson et al. (2020)[2], including a results section.
- **Table S1** – Overview of the study sites sorted by region and latitude, including the availability of cloud-free satellite images with visible breaking waves, the ROI coordinates (in °), and the start and end dates of the X_b time series.
- **Figure S3** – Sampling robustness of the X_b dataset: seasonal distribution of breaking-image acquisitions and temporal resolution (Δt), ordered by region and decreasing latitude over the 2018-2025 period.
- **Table S2** – Source locations of the offshore wave data (H_s, T_p) reported in the literature for each site.
- **Table S3 and Figure S4** – Analysis domain and transect configuration for each study site. Coordinates are given as [latitude; longitude] in decimal degrees (°), together with a schematic representation of the transect setup.
- **Figure S5** – Seasonal comparison between slopes derived from Sentinel-2 imagery and in situ measurements of beach-face and nearshore slopes at four sites (Torrey Pines, Collaroy, Narrabeen and Duck), including a results section.
- **Figure S6** – Satellite-based beach state classification scheme using X_b thresholds, illustrated with representative satellite examples.
- **Figure S7** – Challenges in capturing alongshore variability of offshore breaking using X_b.
- **Figure S8** – Stability of X_b thresholds across different percentile choices.
- **Figure S9** – Influence of tidal range on the proxy X_b and its variability, including the description of the sites and associated results across tidal regimes.

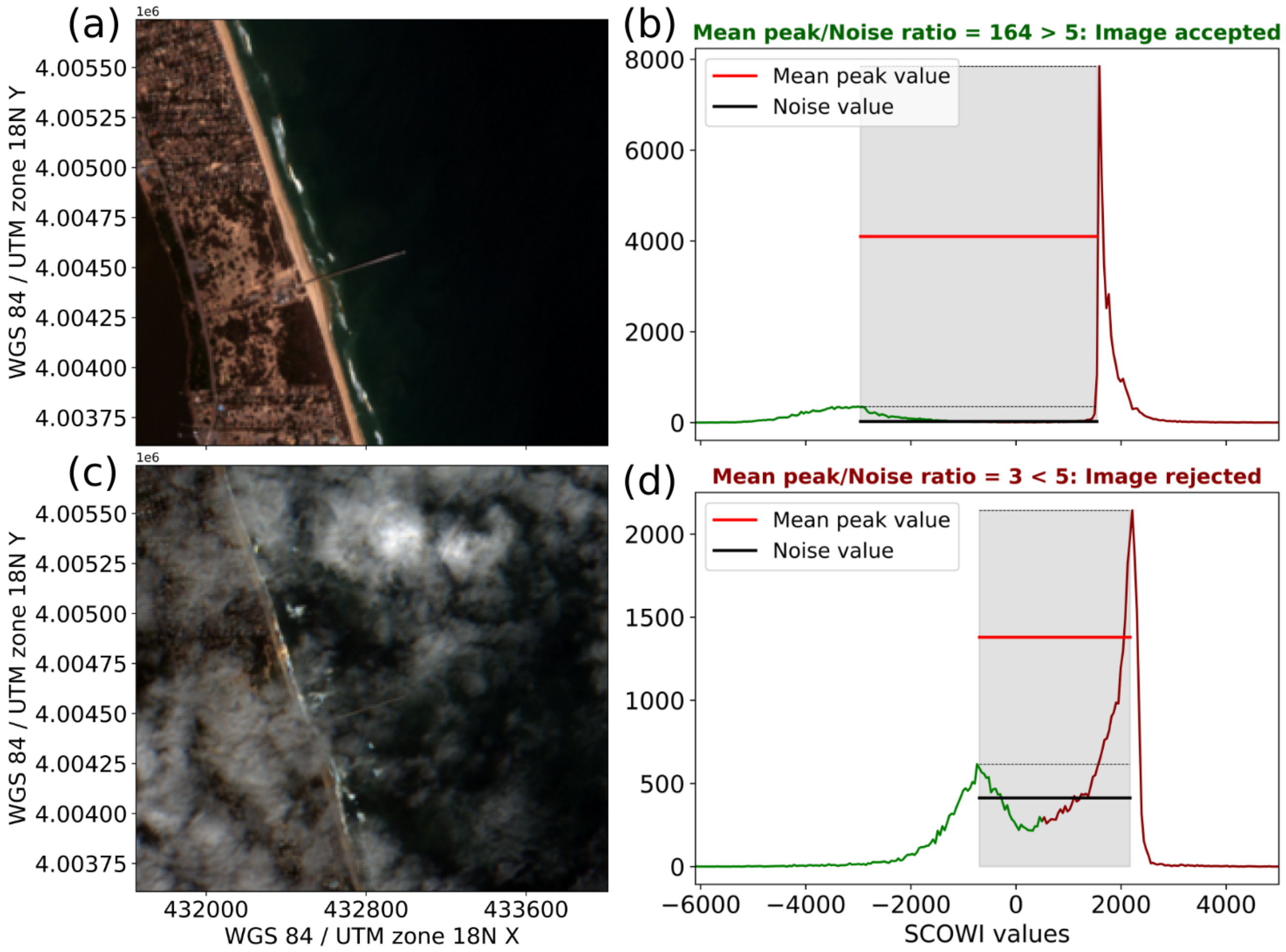

Figure S1. Image filtering based on cloud coverage at FRF-site, modified after[1]. The Sentinel-2 acquisition is shown both **(a)** without and **(c)** with cloud coverage, accompanied by the corresponding SCoWI value histograms **(b)** and **(d)**. The mean peak value (solid red line) represents the average of occurrences at both the land-pixel mode peak and the sea-pixel mode peak. The noise value (solid black line) represents the average of all SCoWI values occurring between the two peaks. If the ratio between the mean peak and the noise value falls below 5, the image is discarded.

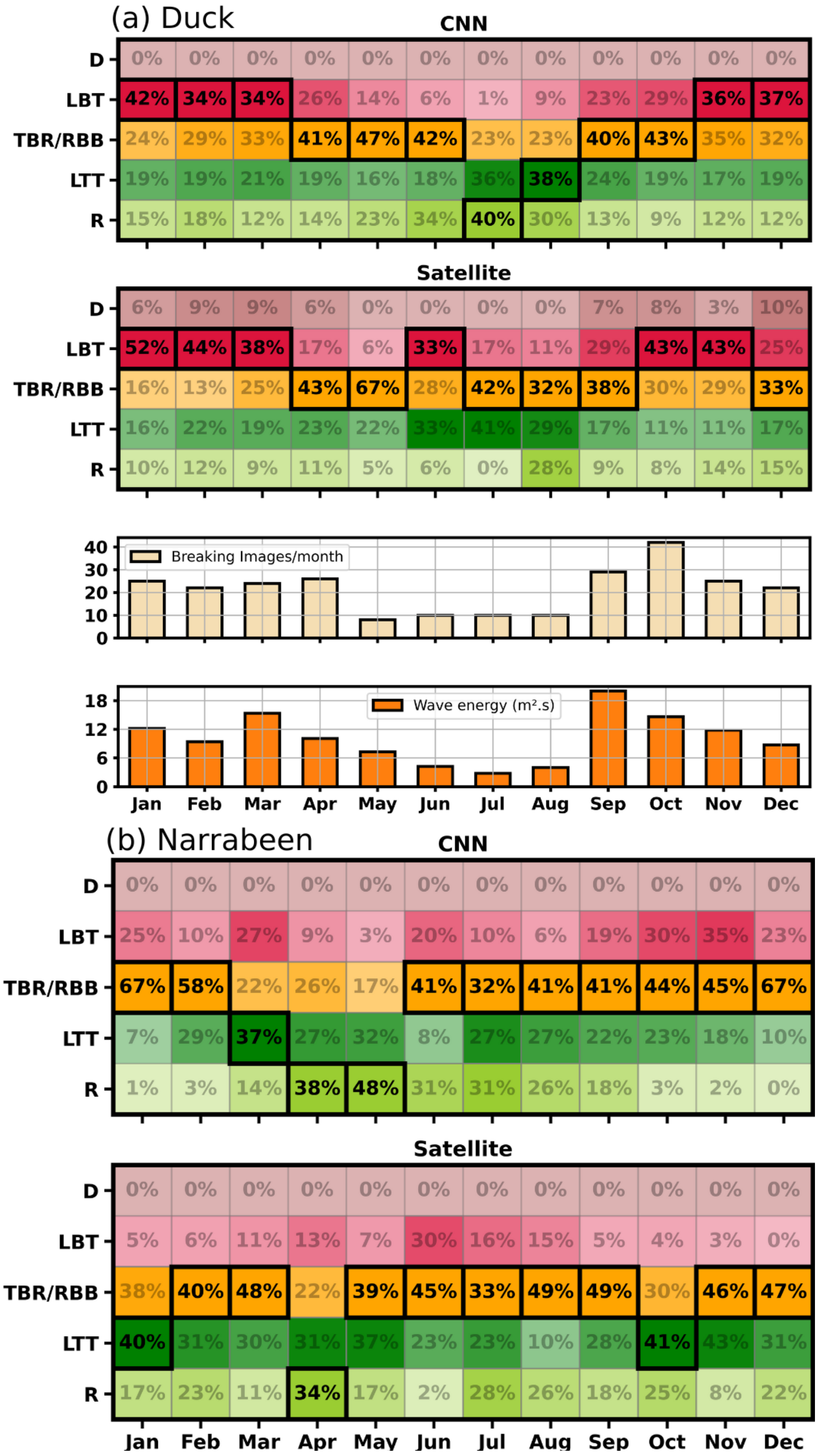

Figure S2. Seasonal cycles of beach state distributions at **(a)** Duck and **(b)** Narrabeen: comparison between satellite-derived X_b classifications (Sentinel-2) and CNN-based classifications from Ellenson et al. (2020), including the influence of limited breaking-wave detection during summer months at Duck.

Direct in situ time series of beach states are not available for the Sentinel-2 period, preventing a straightforward validation of our approach. As an alternative, we used the CNN-based classifications of Ellenson et al. (2020) as a reference[2]; their study applied a convolutional neural network to ARGUS video imagery at Duck (1987-2015) and Narrabeen (2004-2018). We could have attempted to validate using the Narrabeen time series between 2015 and 2018, but our time series start in 2018 because only Sentinel-2A was available between 2015 and 2018, providing very few images; this limited sampling would not allow for a consistent comparison. Figure **S2** shows the comparison of the seasonal cycles between the CNN reference and the satellite-derived X_b classification, at **(a)** Duck and **(b)** Narrabeen. To evaluate the performance of satellite-derived beach state distributions, monthly proportions of each beach state were calculated over a seasonal cycle and compared to the CNN reference. For Duck, the satellite-derived beach state distributions show a mean absolute error (MAE) of 8% and a Pearson correlation coefficient of 0.76 with the CNN reference, indicating a relatively good agreement both in terms of absolute proportions and in capturing temporal variations. The satellite-derived distributions generally capture the expected seasonal trends, with more dissipative states during energetic winter months and more reflective or intermediate states during the calmer summer period. However, during summer (June-July-August), X_b tends to overestimate the beach state. This is due to the limited number of breaking wave images available during summer: lower wave energy often prevents breaking, and when waves do break, they may occur during storm day and over an inactive summer sandbar, and predict an overestimation of the beach state. Figure **S2(a)** provides seasonal cycle of both the number of images where breaking is detected and a simplified proxy for wave energy flux (expressed as $H_s^2 \times T_p$). This quantity is proportional to wave power but does not include physical constants such as ρ (water density) or g (gravitational acceleration), and is therefore used here only to capture relative variations in wave energy throughout the year. The figure confirms that at Duck, few breaking waves are captured in summer, explaining the overestimation of the beach state during this period. For Narrabeen, the MAE is slightly higher at 11% with a correlation of 0.655, suggesting that the satellite still captures the general trends but with larger deviations from the CNN-based distributions. While the CNN accurately captures the transition from intermediate to more reflective states between March and May, this seasonal shift is less clearly resolved by the satellite-derived X_b. Nevertheless, the stability toward TBR/RBB states for the rest of the year is well captured by the satellite.

City	Country	Start date	End date	Cloud-free images % (N)	Breaking images % (N)	Lon_{min}	Lon_{max}	Lat_{min}	Lat_{max}
**Northern Europe (NE)**									
Staengehus	Denmark	2015-08-09	2025-02-23	55 (807)	19 (150)	11.9752	12.0341	56.0218	56.0388
Skallingen	Denmark	2015-07-06	2025-02-27	50 (429)	23 (100)	8.2301	8.2992	55.4753	55.5012
Druridge Bay	UK	2016-02-10	2025-02-03	35 (337)	25 (86)	-1.5863	-1.5176	55.2259	55.3105
Benone	Ireland	2015-12-11	2025-02-15	30 (247)	32 (78)	-6.9038	-6.8119	55.1538	55.1917
Egmond	Netherlands	2015-07-06	2025-02-23	45 (209)	35 (73)	4.6014	4.6243	52.6109	52.6396
Perran Porth	UK	2015-07-25	2025-02-23	35 (586)	61 (361)	-5.1698	-5.1399	50.3454	50.3800
Truc Vert	France	2015-08-18	2025-02-07	57 (306)	86 (264)	-1.2844	-1.2200	44.6957	44.7600
**North America (NA)**									
Elwha	US	2015-11-26	2025-02-16	59 (283)	23 (66)	-123.6005	-123.5690	48.1357	48.1459
Newport	US	2015-10-04	2025-02-05	100 (247)	97 (240)	-124.0832	-124.0559	44.6461	44.6711
Duck	US	2015-11-13	2025-02-02	74 (634)	59 (376)	-75.7602	-75.7337	36.1749	36.1941
Torrey Pines	US	2015-08-03	2025-02-15	67 (724)	87 (630)	-117.2754	-117.2452	32.8819	32.9072
Ensenada	Mexico	2015-08-10	2025-02-01	70 (397)	90 (357)	-116.6335	-116.6038	31.7837	31.8274
**Mediterranean (MED)**									
Racou	France	2015-10-01	2025-02-15	67 (600)	5 (32)	3.0512	3.0656	42.5363	42.5426
Ebro	Spain	2017-07-05	2025-02-14	58 (599)	4 (22)	0.7742	0.8474	40.6466	40.6753
Cala Millor	Spain	2015-10-01	2025-02-07	69 (796)	7 (54)	3.3838	3.3966	39.5879	39.6055
Armação de Pera	Portugal	2016-05-10	2025-02-09	69 (379)	10 (39)	-8.3533	-8.3190	37.0848	37.1020
Fnideq	Morocco	2016-02-17	2025-02-30	63 (736)	7 (50)	-5.3567	-5.3408	35.8130	35.8296
**Asia (AS)**									
Hasaki	Japan	2016-01-24	2025-02-07	53 (547)	74 (407)	140.7511	140.7886	35.8281	35.8527
Ganpatipule	India	2016-07-14	2025-02-13	82 (690)	9 (62)	73.2569	73.2700	17.1354	17.1466
**Africa (AF)**									
Saint Louis	Senegal	2016-01-14	2025-02-20	79 (434)	58 (250)	-16.5205	-16.5017	16.0178	16.0399
Grand Popo	Benin	2015-10-22	2025-02-23	66 (537)	13 (69)	1.7306	1.7651	6.2495	6.2661
**South America (SA)**									
Bocatocino	Colombia	2015-11-24	2025-02-20	72 (659)	37 (243)	-75.2082	-75.1923	10.8148	10.8272
Mariscal	Brazil	2017-04-14	2025-02-18	61 (474)	39 (183)	-48.5045	-48.4780	-27.2033	-27.1648
Mataquito	Chile	2015-08-18	2025-02-19	70 (601)	45 (270)	-72.2133	-72.1793	-34.9791	-34.9503
**Oceania (OC)**									
Cable	Australia	2016-03-20	2025-02-27	84 (458)	4 (17)	122.1960	122.2251	-17.9355	-17.8798
Harbour	Australia	2015-12-18	2025-02-12	64 (381)	26 (100)	149.2146	149.2293	-21.1472	-21.1150
Nine Mile	Australia	2015-12-05	2020-02-26	45 (304)	70 (213)	150.7791	150.8014	-22.9183	-22.8229
Gold Coast	Australia	2015-09-30	2025-02-24	61 (326)	86 (279)	153.4278	153.4419	-28.0170	-27.9916
Pearl	Australia	2016-01-01	2025-02-03	62 (610)	97 (593)	151.3059	151.3185	-33.5454	-33.5368
Palm	Australia	2015-10-20	2025-02-13	67 (650)	73 (477)	151.3239	151.3372	-33.6005	-33.5795
Narrabeen	Australia	2015-10-20	2025-02-04	66 (634)	73 (463)	151.2988	151.3124	-33.7324	-33.7040
Collaroy	Australia	2015-10-20	2025-02-04	66 (634)	43 (271)	151.2988	151.3124	-33.7324	-33.7040
Seven Mile	Australia	2015-10-20	2025-02-18	51 (456)	83 (378)	150.7534	150.7757	-34.8336	-34.8092
Goolwa	Australia	2015-10-28	2025-02-18	49 (511)	90 (461)	138.7428	138.7802	-35.5399	-35.5131
Pedro	Australia	2015-09-03	2025-02-08	70 (630)	47 (296)	150.1528	150.1671	-35.9559	-35.9310
Tairua	New Zealand	2015-08-05	2025-02-12	52 (440)	61 (268)	175.8526	175.8732	-36.9990	-36.9857
Canterbury	New Zealand	2015-12-31	2025-02-08	50 (231)	39 (91)	171.9232	171.9908	-44.0049	-43.9732

Table S1. Overview of study sites sorted by region and then with latitude, with availability of cloud-free, breaking-wave satellite images, and ROI coordinates (in °). The start and end dates of the X_b time series are also provided for each site.

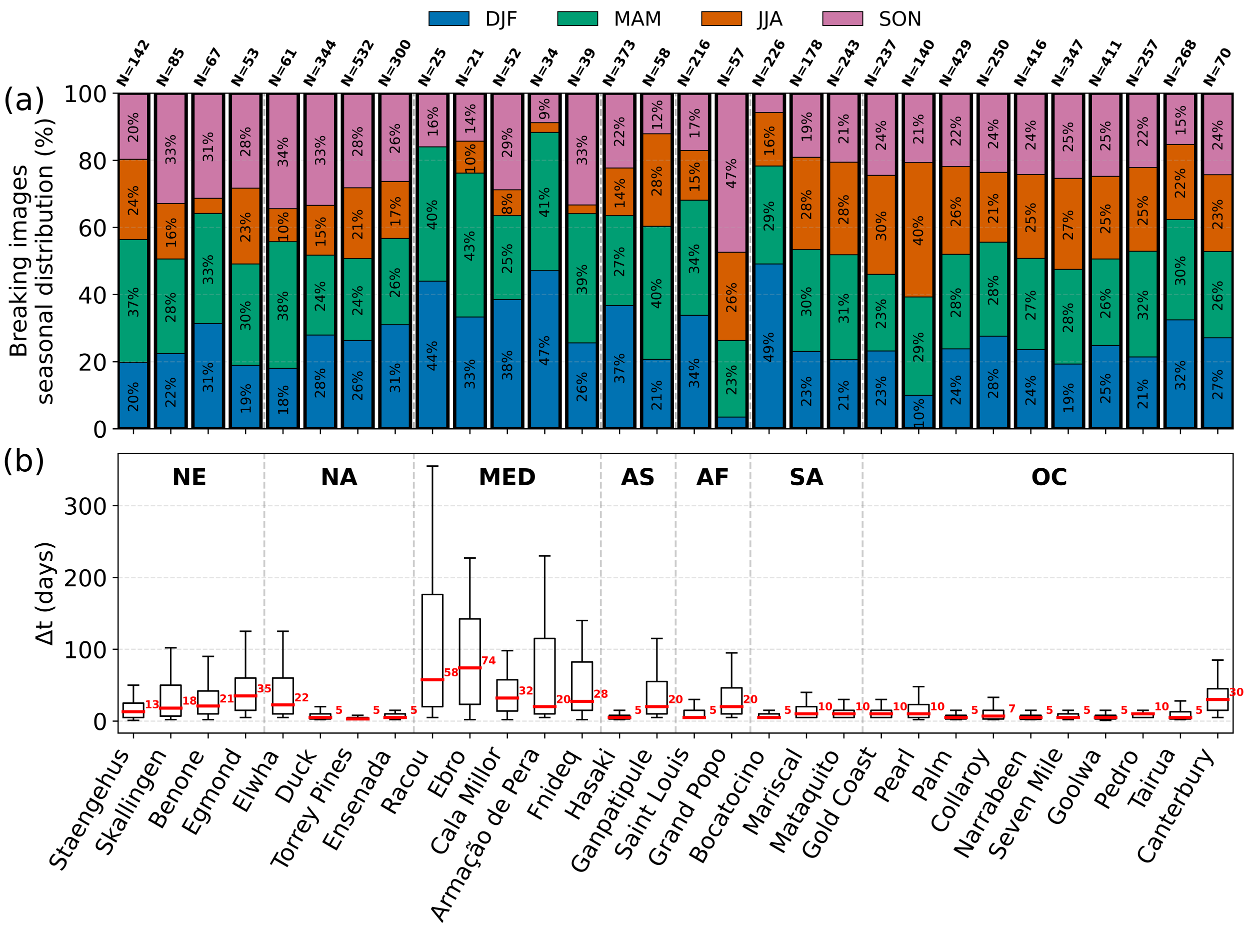

Figure S3. Sampling robustness of the X_b dataset: seasonal distribution of breaking-image acquisitions and temporal resolution (Δt), ordered by region and decreasing latitude (from *instantaneous signal* during 2018-2025 period).

Site	Country	lon (°)	lat (°)	depth (m)
		**NE**		
Staengehus[3]	Denmark	11.91	52.03	-
Skallingen[3,4]	Denmark	8.20	55.31	-
Benone[5]	Ireland	-9.18	54.66	-
Egmond[6]	Netherlands	4.32	52.62	-
		**NA**		
Elwha[7]	US	-124.72	48.49	-
Duck[8]	US	-75.69	36.20	-
Torrey Pines[9]	US	-117.16	32.56	21
Ensenada[10]	Mexico	-116.62	31.80	24
		**MED**		
Racou[11]	France	3.16	42.48	50
Ebro[12]	Spain	0.89	40.72	50
Cala Millor[13]	Spain	3.39	39.59	17
Armaçao de Pera[14]	Portugal	-7.88	36.90	-
Fnideq[15]	Morocco	-5.31	35.92	-
		**AS**		
Hasaki[16]	Japan	140.75	35.8	24
Ganpatipule[17]	India	16.99	73.23	15
		**AF**		
Saint Louis[18]	Senegal	-17	16.5	-
Grand Popo[19]	Benin	1.77	5.97	-
		**SA**		
Bocatocino[20,21]	Colombia	-74.88	11.22	-
Mariscal[22]	Brazil			
Mataquito[23]	Chile	-34.96	-72.19	11.6
		**OC**		
Gold Coast[24,25]	Australia	153.44	-27.96	18
Pearl[3]	Australia	151.49	-33.77	-
Palm[26]	Australia	151.83	-33.79	80
Narrabeen[27]	Australia	151.42	-33.78	80
Collaroy[27]	Australia	151.42	-33.78	80
Seven Mile[28]	Australia	151.03	-34.98	80
Goolwa[28–30]	Australia	137.61	-37.12	80
Pedro[31]	Australia	150.23	-35.93	-
Tairua[32]	New Zealand	175.11	-35.92	-
Canterbury[33,34]	New Zealand	172.5	-44.2	74

Table S2. Source locations of the wave data (H_s, T_p) reported in the literature for each site. Coordinates are given in decimal degrees (°) and depth corresponds to the measurement depth when specified.

Site	Country	Beach state	L (m)	l (m)	e (m)	n	A coordinates	B coordinates
Canterbury[33,34]	New Zealand	R	2000	700	30	67	[-43.98364;17.96677]	[-43.99284;171.94582]
Fnideq[15]	Morocco	R	1600	650	30	53	[35.82820;-5.35350]	[35.81397;-5.35288]
Racou[11]	France	R	400	275	30	14	[42.53987;3.05376]	[42.53700;3.05661]
Pearl[3]	Australia	R	750	520	30	25	[-33.53783;151.31095]	[-33.54388;151.30697]
Harbour[35]	Australia	R	2580	520	30	86	[-21.11989;149.22186]	[-21.14304;149.21940]
Grand Popo[19]	Benin	LTT	3100	900	30	103	[6.26362;1.76014]	[6.25775;1.73251]
Elwha[7]	US	LTT	870	530	30	29	[48.14176;-123.57194]	[48.13689;-123.58111]
Collaroy[27]	Australia	LTT	600	750	30	20	[-33.72649;151.29958]	[-33.73178;151.30144]
Druridge Bay[35,36]	UK	LTT	5500	1840	30	183	[55.29110;-1.57210]	[55.24270;-1.55180]
Nine Mile[35]	Australia	LTT	1920	1020	30	64	[-22.85171;150.79199]	[-22.86893;150.78928]
Narrabeen[27]	Australia	TBR-RBB	1530	780	30	51	[-33.70578;151.30475]	[-33.71915;151.29933]
Gold Coast[24,25]	Australia	TBR-RBB	2530	800	30	84	[-27.99347;153.43028]	[-28.01629;153.43290]
Duck[8]	US	TBR-RBB	1870	730	30	62	[36.19136;-75.75524]	[36.17538;-75.861]
Pedro[31]	Australia	TBR-RBB	1940	720	30	65	[-35.93460;150.15847]	[-35.95179;150.15623]
Tairua[32]	New Zealand	TBR-RBB	1090	485	30	36	[-36.98930;175.85550]	[-36.99669;175.86241]
Palm[26]	Australia	TBR-RBB	1920	700	30	64	[-33.58185;151.32927]	[-33.59886;151.32469]
Ganpatipule[17]	India	TBR-RBB	830	780	30	28	[17.14585;73.26616]	[17.13862;73.26836]
Armaçao de Pera[14]	Portugal	TBR-RBB	1750	700	30	58	[37.09862;-8.34895]	[37.09083;-8.33181]
Cala Millor[13]	Spain	TBR-RBB	1540	630	30	51	[39.60255;3.38537]	[39.58866;3.38526]
Truc Vert[37]	France	TBR-RBB	4120	2000	30	137	[44.74950;-1.24030]	[44.71310;-1.24660]
Hasaki[16]	Japan	LBT	2380	780	30	79	[35.84692;140.75396]	[35.82887;140.76787]
Saint Louis[18]	Senegal	LBT	2180	870	30	73	[16.03842;-16.50854]	[16.01857;-16.50914]
Egmond[6]	Netherlands	LBT	2780	1240	30	93	[52.63672;4.62441]	[52.61198;4.61999]
Torrey Pines[9]	US	LBT	1950	780	30	65	[32.90425;-117.25429]	[32.88646;-117.25275]
Ensenada[10]	Mexico	LBT	1730	680	30	58	[31.81354;-116.60817]	[31.79796;-116.61105]
Staengehus[3]	Denmark	LBT	1870	780	30	62	[56.03329;12.01650]	[56.02366;11.99213]
Ebro[12]	Spain	LBT	2240	760	30	77	[40.67334;0.82740]	[40.66397;0.80386]
Goolwa[28–30]	Australia	D	2240	780	30	75	[-35.51768;138.75083]	[-35.52300;138.77468]
Seven Mile[28]	Australia	D	2090	680	30	70	[-34.81138;150.76503]	[-34.82868;150.75606]
Bocatocino[20,21]	Colombia	D	1290	800	30	43	[10.81730;-75.19417]	[10.81705;-75.20595]
Mariscal[22]	Brazil	D	3090	725	30	103	[-27.16994;-48.49930]	[-27.19758;-48.49654]
Skallingen[3,4]	Denmark	D	2190	730	30	73	[55.49821;8.25627]	[55.48453;8.28103]
Benone[5]	Ireland	D	2460	800	30	82	[55.17052;-6.90061]	[55.16616;-6.86298]
Mataquito[23]	Chile	D	2730	660	30	91	[-34.95218;-72.18591]	[-34.97679;-72.18474]
Perran Porth[38]	UK	D	2860	1100	30	95	[50.37471;-5.14574]	[50.34960;-5.15450]
Newport[39]	US	D	5010	1130	30	167	[44.67080;-124.05978]	[44.64657;-124.05974]
Cable[35]	Australia	D	2940	1370	30	98	[-17.88366;122.21569]	[-17.91003;122.21221]

Table S3. Analysis domain and transect configuration for each study site. Coordinates are given as [latitude; longitude] in decimal degrees (°).

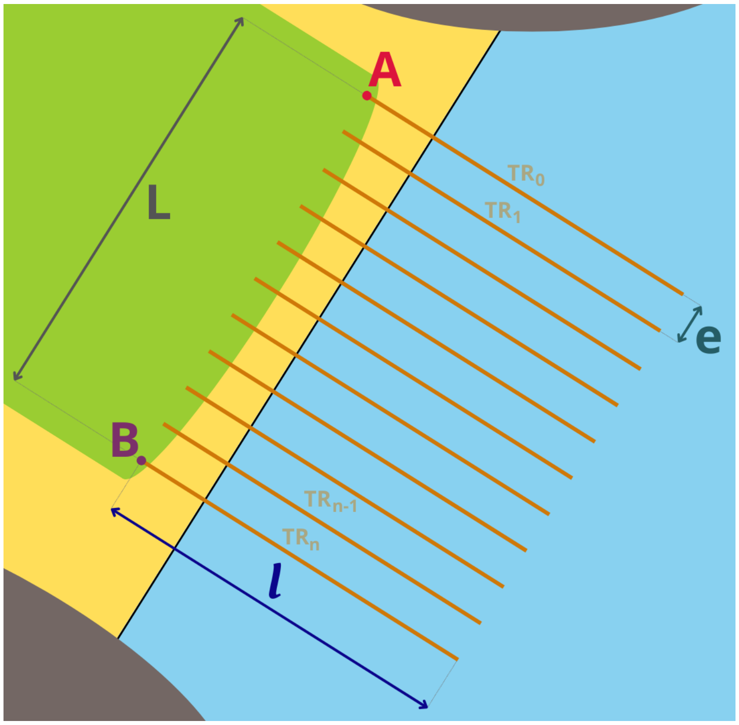

Figure S4. Schematic representation of the analysis domain and cross-shore transect configuration. The alongshore segment is bounded by endpoints A and B, separated by a distance L. Cross-shore transects (TR_0 to TR_n) are generated perpendicular to a baseline digitized along the landward edge of the beach. Transects are spaced every e meters and extend seaward over a length ℓ.

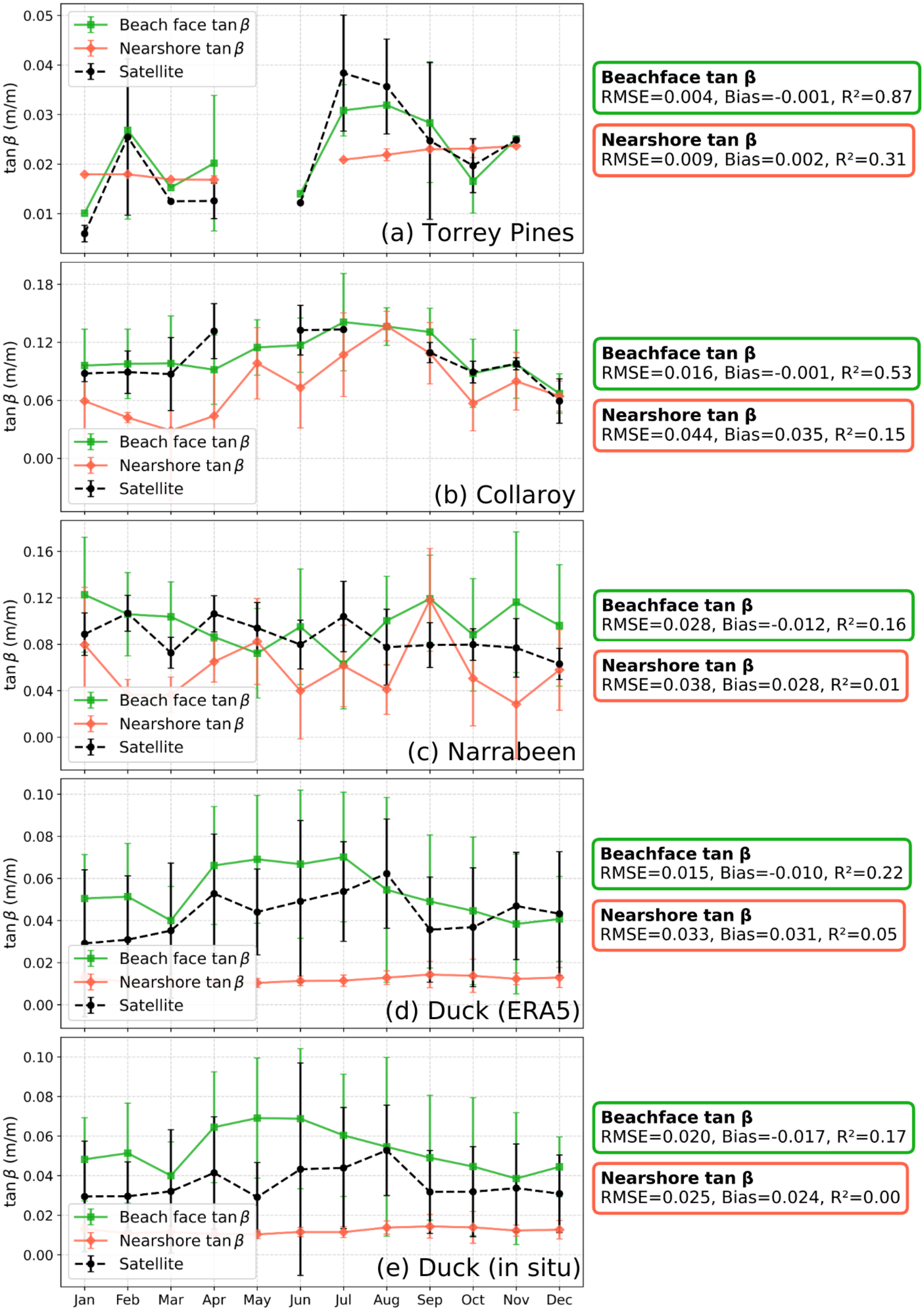

Figure S5. Seasonal comparison of slope from Sentinel-2 and in situ beach face and nearshore slopes at four study sites (Torrey Pines, Collaroy, Narrabeen and Duck), with associated R^2, RMSE, and bias statistics.

To further assess the capability of the X_b indicator, we compared beach slopes derived from in situ topographic profiles with the one estimated from satellite-derived X_b. Two in situ slope metrics were considered: the beach face slope, computed between MSL ± 0.5 m (Mean Sea Level), and the nearshore slope, computed between MSL and depth of closure[40]. In situ slopes were obtained by linear regression of elevation profiles within these vertical windows, after correcting for tidal level and site-specific vertical references to MSL. The topographic profiles were derived from existing datasets: Torrey Pines[9] (vertical reference of 0.774 m), Narrabeen[27] (0 m), and Duck[41] (-0.128 m). Rather than validating the time series point by point, we focused on the seasonal cycle, which provides a robust benchmark of beach slope dynamics, especially given that the periods of available in situ data and Sentinel-2 observations did not perfectly overlap. For each site, monthly means of in situ and satellite slopes were compared using R^2, RMSE, and bias statistics. At Torrey Pines, a longshore bar and trough beach tending towards dissipative conditions, the agreement was strongest for the beach face slope (R^2=0.87, RMSE=0.004, (**a**)). Slopes were steeper in summer ($\sim$0.035–0.04) and flatter in winter ($\sim$0.015–0.02), in phase with satellite estimates. The nearshore slope, by contrast, remained nearly constant throughout the year and was poorly correlated (R^2=0.31). This is consistent with the fact that the nearshore slope, defined between the mean sea level and the depth of closure, represents a more persistent morphological feature shaped by long-term sediment exchanges rather than short-term seasonal variability[40]. At Collaroy, a low tide terrace beach, the beach face slope also exhibited a seasonal cycle consistent with satellite observations (R^2=0.53, (**b**)), with steeper profiles in July (>0.12) and flatter ones in December ($\sim$0.06). Weaker agreement was found on the nearshore slope (R^2=0.15), although some seasonal coherence was preserved during late winter and spring. At Narrabeen, results were less robust: beach face slopes showed only weak correlation with satellite estimates (R^2=0.16), with no clear seasonal cycle. The nearshore slope performed even worse (R^2=0.01), reflecting the site's high morphodynamic variability and spatial complexity; however, the median slope was reasonably well captured. At Duck, the beach face slopes showed a modest seasonal agreement (R^2=0.22), with steeper profiles in spring-early summer ($\sim$0.07 in situ vs. $\sim$0.05 satellite) and flatter slopes in autumn ($\sim$0.04). The nearshore slope was again poorly correlated (R^2=0.05). Using in situ wave data instead of ERA5 did not significantly improve the results. Also, during summer months at Duck, satellite-derived states slightly overestimate the beach state, classifying reflective states as intermediate (Section CNN validation), which naturally results in slightly gentler slopes compared to in situ observations during those months. These findings indicate that X_b is primarily sensitive to the beach face slope, which reflects the morphology of the active breaker zone, rather than the more stable nearshore slope. The monthly standard deviations highlight this behavior: they do not represent transect-to-transect variability, but instead the intra-month variability of the active profile. This variability is controlled by short-term fluctuations in wave forcing, storm–recovery cycles, and the tidal modulation of the breaker zone. Months with higher standard deviations thus correspond to more dynamic conditions, whereas low values reflect more stable hydrodynamic and morphodynamic regimes. Overall, the seasonal cycles of beach face slopes at Torrey Pines and Collaroy are well captured by the satellite-derived indicator, even though the agreement is weaker at Narrabeen and Duck. This observation highlights its potential value for studying nearshore morphodynamics, although further validation across contrasting settings is required.

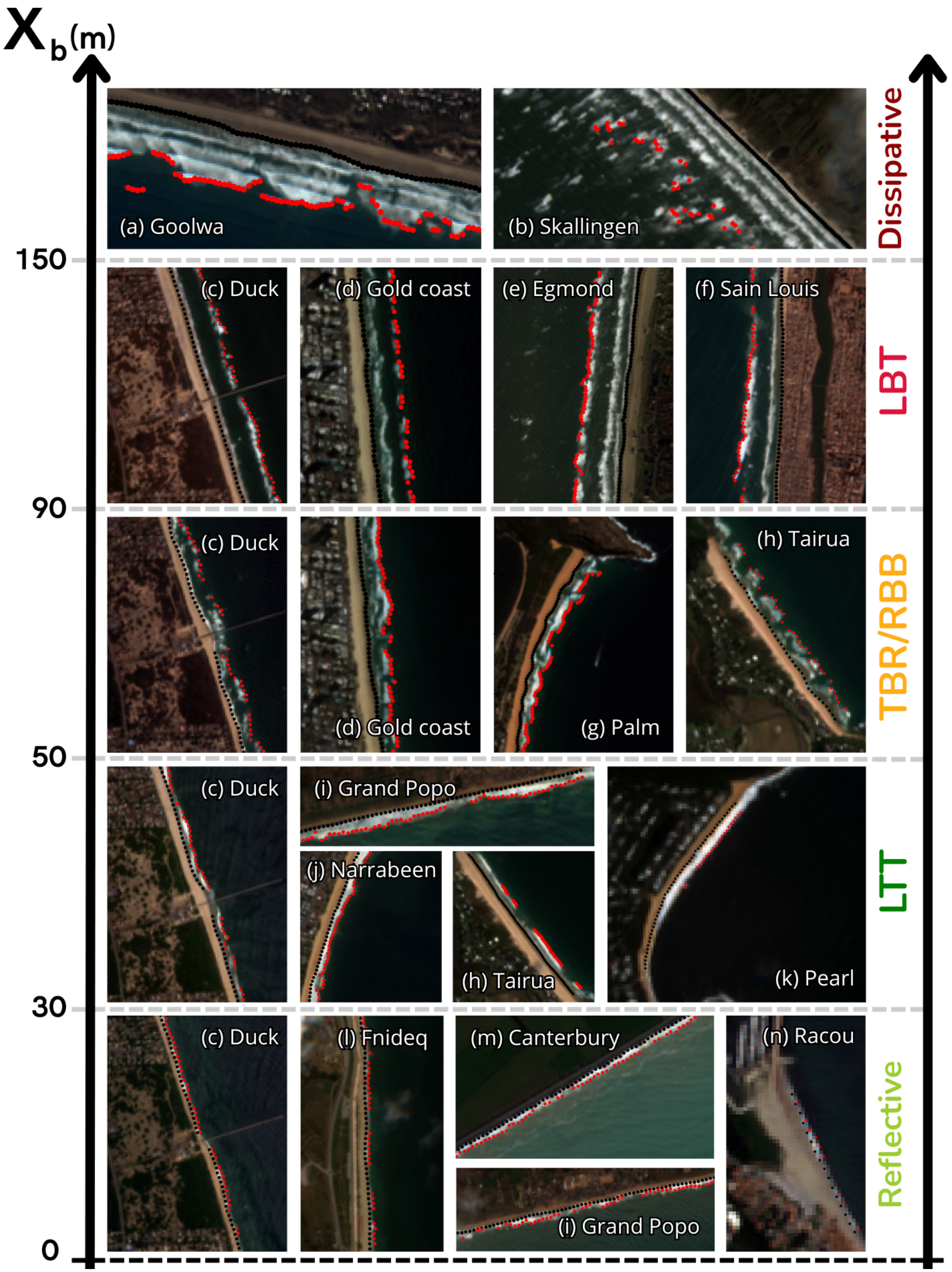

Figure S6. Satellite-based beach state classification scheme using X_b thresholds. Example sites are indicated as letters: **(a)** Goolwa (D), **(b)** Skallingen (D), **(c)** Duck (LBT,TBR/RBB,LTT,R), **(d)** Gold Coast (LBT,TBR/RBB), **(e)** Egmond (LBT), **(f)**Saint Louis (LBT), **(g)** Palm (TBR/RBB), **(h)** Tairua (TBR/RBB), **(i)** Grand Popo (LTT, R), **(j)** Narrabeen (LTT), **(k)** Pearl (LTT), **(l)** Fnideq (R), **(m)** Canterbury (R), **(n)** Racou (R).

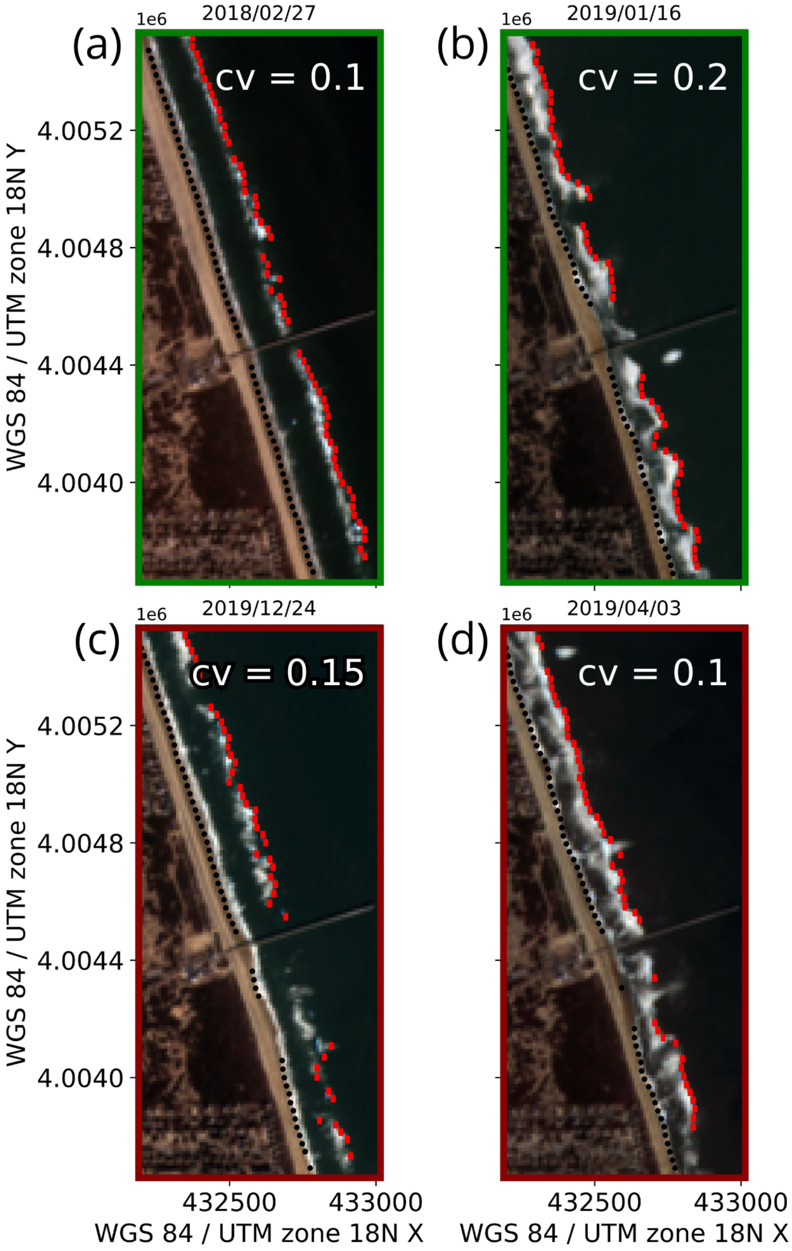

Figure S7. Challenges in capturing alongshore variability of offshore breaking using X_b: ideal versus (green frames) ambiguous cases (dark red frames).

Illustration of the challenges in using the coefficient of variation (CV) of the most offshore breaking position (X_b) as a proxy for alongshore bar morphology. The CV is computed as the standard deviation of X_b along all transects divided by its mean, providing a measure of alongshore variability. Based on extensive inspection of satellite images, we observed that CV ≤ 0.1 generally indicates a linear bar (LBT), whereas CV > 0.1 corresponds to more three-dimensional forms (RBB, TBR). Panels **(a)** and **(b)** show ideal cases: a linear bar type (LBT) with uniform breaking along transects (CV ≈ 0.1), and a three-dimensional bar (RBB) (CV ≈ 0.2). Panels **(c)** and **(d)** illustrate more ambiguous situations where CV alone may misrepresent the underlying morphology despite quasi-uniform breaking along transects. In **(c)**, a linear bar exhibits CV ≈ 0.15 due mostly to the instantaneous variability of breaking, while in **(d)** the bar is three-dimensional (clearly visible by the presence of rip channels/currents), yet CV ≈ 0.1 because the offshore breaking appears aligned across transects. These examples highlight that, unlike video-based studies where wave breaking can be averaged over 10-min, satellite imagery captures only instantaneous snapshots and limits the ability of simple metrics like CV to reliably detect three-dimensional bar structures, highlighting the need for more sophisticated approaches to capture alongshore variability.

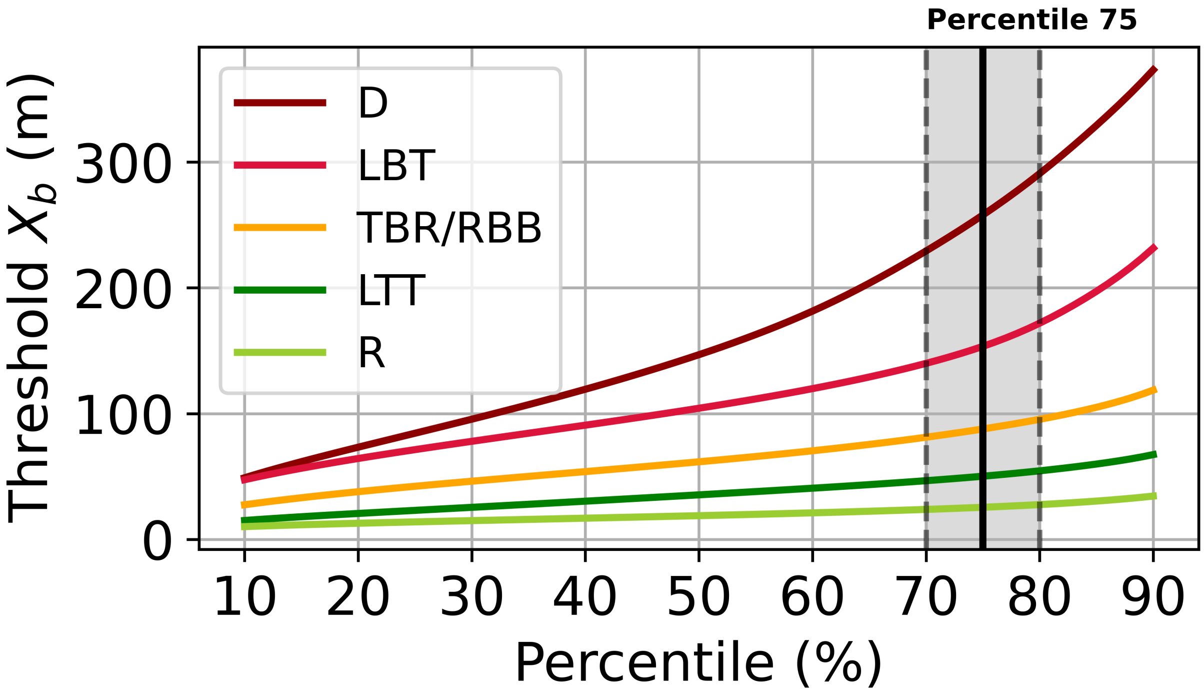

Figure S8. Stability of X_b thresholds across percentile choices.

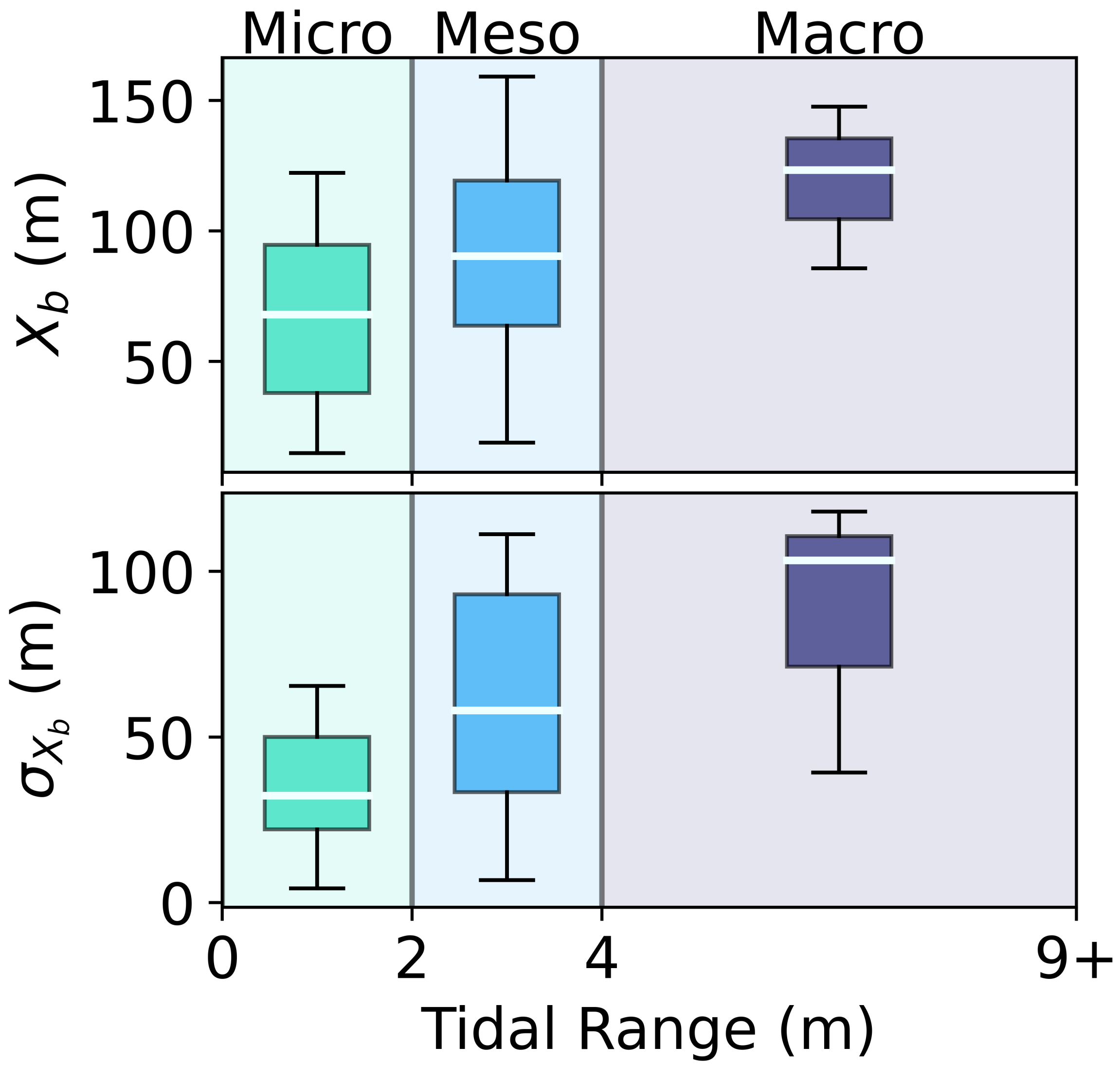

Figure S9. Influence of tidal range on the proxy X_b and its variability.

These include Cable Beach (RTR = 12, $\Omega = 2.5$,[35]), Druridge Bay (RTR = 9, $\Omega = 0.6$,[35,36]), Harbour Beach (RTR = 8, $\Omega = 1.5$,[35]), Nine Mile Beach (RTR = 5–6, $\Omega = 3$–3.5,[35]), Perran Porth (RTR = 3.2, $\Omega \approx 1$,[38]), Newport (MSR = 2.5 m,[39]), and Truc Vert (MSR=5m,[37]), a double-barred intermediate beach. The morphological characteristics of these beaches are diverse: Cable Beach is an ultra-dissipative site that features subdued ridges and runnels around neap high tides while reflective conditions can produce cusps at spring high tide; Druridge Bay and Nine Mile Beach exhibit both low-tide terrace (LTT) profiles; however, Druridge Bay is characterized by ridges and runnels, whereas Nine Mile Beach displays low-tide bar/rip morphology. Harbour Beach displays steep high-tide beach faces with distinct slope breaks. Perranporth and Newport are dissipative with wide surf zones and offshore bars; and Truc Vert shows complex morphology with a TBR oblique inner bar and RBB outer bar. However, beyond this morphological diversity, tidal modulation introduces a high degree of variability in wave breaking patterns, which complicates the interpretation of breaker-derived indicators such as X_b. Taking the example of Truc Vert, wave breaking is not fixed but depends on tidal stage: at low tide, waves may primarily break on the outer bar and at the waterline (confounded with the intertidal oblique TBR bar), whereas at high tide, breaking can occur simultaneously on the outer bar (though less frequently), the inner bar, and the waterline located farther landward. In such cases, the most offshore breaker line, commonly used as an indicator of beach state, becomes ambiguous, as it no longer reflects a single, dominant morphology. A similar issue arises on LTT beaches, which, in macrotidal contexts, live up to their name. At high tide, the steep upper beach behaves reflectively, whereas at low tide the gentle terrace exhibits a dissipative character. Assigning a single, median beach state therefore becomes problematic, since the effective state oscillates strongly with the tide. This complexity is further illustrated by our analysis of X_b across tidal ranges. Using a whisker plot of median X_b values for all sites, grouped by tidal range categories (micro: 0–2 m, meso: 2–4 m, macro: >4 m), Figure S9 illustrates the influence of tidal range on X_b. The top whisker plot shows the distribution of site-specific median X_b values within tidal range category (micro, meso, macro). The median X_b increases at higher tidal range, reflecting the general surf zone broadening with increased tidal excursions. The interquartile range (IQR = P25–P75) is larger for micro- and meso-tidal sites and decreases for macro-tidal sites, which means that macro-tidal beaches have more organized morphodynamic configurations (e.g., more stable bars and dominant dissipation processes), and thus lower variability in median X_b among sites. On the bottom, the median of the standard deviation of X_b within each site also grouped by their tidal range is shown, highlighting temporal variability. Here, meso- and macro-tidal sites exhibit larger IQR, suggesting that the instantaneous breaking location fluctuates more over time at sites with larger tidal ranges. This result highlights a fundamental limitation: thresholds derived from X_b for wave-dominated beaches cannot be directly applied to tide-modified or tide-dominated systems without accounting for tidal influence. Several approaches can help address this limitation. One potential solution is to separate the X_b time series into high-tide and low-tide subsets, assigning distinct beach states for each tidal phase. However, splitting the dataset reduces the number of observations per subset, making it difficult to define robust thresholds with current satellite revisit times. Future high-frequency satellite missions, such as VENμS or upcoming ones, could enable sufficient temporal coverage to resolve beach-state dynamics at both tidal extremes. As a perspective, *eo-tides* could be used to resolve the intertidal zone more precisely[42,43], allowing X_b to be analyzed separately for different tidal phases. This approach may enable tidal-aware beach-state thresholds and better capture the dynamics of tide-modified systems.

References

1. Graffin, M. *et al.* Waterline responses to climate forcing along the North American West Coast. *Commun. Earth & Environ.* **6**, 444, DOI: 10.1038/s43247-025-02414-x (2025).

2. Ellenson, A. N., Simmons, J. A., Wilson, G. W., Hesser, T. J. & Splinter, K. D. Beach State Recognition Using Argus Imagery and Convolutional Neural Networks. *Remote. Sens.* **12**, 3953, DOI: 10.3390/rs12233953 (2020). Number: 23.

3. Aagaard, T., Greenwood, B. & Hughes, M. Sediment transport on dissipative, intermediate and reflective beaches. *Earth-Science Rev.* **124**, 32–50, DOI: 10.1016/j.earscirev.2013.05.002 (2013).

4. Aagaard, T. & Kroon, A. Decadal behaviour of a washover fan, Skallingen Denmark. *Earth Surf. Process. Landforms* **44**, 1755–1768, DOI: 10.1002/esp.4610 (2019). _eprint: https://onlinelibrary.wiley.com/doi/pdf/10.1002/esp.4610.

5. Jackson, D. W. T., Cooper, J. A. G. & del Rio, L. Geological control of beach morphodynamic state. *Mar. Geol.* **216**, 297–314, DOI: 10.1016/j.margeo.2005.02.021 (2005).

6. Aagaard, T. *et al.* Intertidal beach change during storm conditions; Egmond, The Netherlands. *Mar. Geol.* **218**, 65–80, DOI: 10.1016/j.margeo.2005.04.001 (2005).

7. Miller, I. M., Warrick, J. A. & Morgan, C. Observations of coarse sediment movements on the mixed beach of the Elwha Delta, Washington. *Mar. Geol.* **282**, 201–214, DOI: 10.1016/j.margeo.2011.02.012 (2011).

8. Lippmann, T. C. & Holman, R. A. The spatial and temporal variability of sand bar morphology. *J. Geophys. Res. Ocean.* **95**, 11575–11590, DOI: 10.1029/JC095iC07p11575 (1990). _eprint: https://onlinelibrary.wiley.com/doi/pdf/10.1029/JC095iC07p11575.

9. Ludka, B. C. *et al.* Sixteen years of bathymetry and waves at San Diego beaches. *Sci. Data* **6**, 161, DOI: 10.1038/s41597-019-0167-6 (2019).

10. Ruiz de Alegría-Arzaburu, A., Vidal-Ruiz, J. A., García-Nava, H. & Romero-Arteaga, A. Seasonal morphodynamics of the subaerial and subtidal sections of an intermediate and mesotidal beach. *Geomorphology* **295**, 383–392, DOI: 10.1016/j.geomorph.2017.07.021 (2017).

11. Aleman, N., Robin, N., Certain, R., Anthony, E. J. & Barusseau, J. P. Longshore variability of beach states and bar types in a microtidal, storm-influenced, low-energy environment. *Geomorphology* **241**, 175–191, DOI: 10.1016/j.geomorph.2015.03.029 (2015).

12. Guillén, J. & Palanques, A. Longshore bar and trough systems in a microtidal, storm-wave dominated coast: The Ebro Delta (Northwestern Mediterranean). *Mar. Geol.* **115**, 239–252, DOI: 10.1016/0025-3227(93)90053-X (1993).

13. Fernández-Mora, A., Criado-Sudau, F. F., Gómez-Pujol, L., Tintoré, J. & Orfila, A. Ten years of morphodynamic data at a micro-tidal urban beach: Cala Millor (Western Mediterranean Sea). *Sci. Data* **10**, 301, DOI: 10.1038/s41597-023-02210-2 (2023).

14. Pinto, C. A., Taborda, R., Andrade, C. & Teixeira, S. B. Seasonal and Mesoscale Variations at an Embayed Beach (Armação De Pera, Portugal). *J. Coast. Res.* 118–122 (2009).

15. Anfuso, G., Martínez del Pozo, J. A., Nachite, D., Benavente, J. & Macias, A. Morphological characteristics and medium-term evolution of the beaches between Ceuta and Cabo Negro (Morocco). *Environ. Geol.* **52**, 933–946, DOI: 10.1007/s00254-006-0535-3 (2007).

16. Kuriyama, Y., Ito, Y. & Yanagishima, S. Medium-term variations of bar properties and their linkages with environmental factors at Hasaki, Japan. *Mar. Geol.* **248**, 1–10, DOI: 10.1016/j.margeo.2007.10.006 (2008).

17. Sharma, S., , K., V. Sanil, & Gowthaman, R. Textural characteristics and morphosedimentary environment of foreshore zone along Ganpatipule, Maharashtra, India. *Mar. Georesources & Geotechnol.* **35**, 887–894, DOI: 10.1080/1064119X.2016.1259697 (2017). _eprint: https://doi.org/10.1080/1064119X.2016.1259697.

18. Ndour, A. *et al.* On the Natural and Anthropogenic Drivers of the Senegalese (West Africa) Low Coast Evolution: Saint Louis Beach 2016 COASTVAR Experiment and 3D Modeling of Short Term Coastal Protection Measures. *J. Coast. Res.* **95**, 583–587, DOI: 10.2112/SI95-114.1 (2020).

19. Abessolo Ondoa, G. *et al.* Beach Response to Wave Forcing from Event to Inter-Annual Time Scales at Grand Popo, Benin (Gulf of Guinea). *Water* **9**, 447, DOI: 10.3390/w9060447 (2017). Number: 6.

20. Guerrero, A. M., Otero, L., Ospino, S. & Cueto, J. Interactions between Hydrodynamic Forcing, Suspended Sediment Transport, and Morphology in a Microtidal Intermediate-Dissipative Beach. *J. Mar. Sci. Eng.* **12**, 1141, DOI: 10.3390/jmse12071141 (2024). Number: 7.

21. Otero, L. J., Ortiz-Royero, J. C., Ruiz-Merchan, J. K., Higgins, A. E. & Henriquez, S. A. Storms or cold fronts: what is really responsible for the extreme waves regime in the Colombian Caribbean coastal region? *Nat. Hazards Earth Syst. Sci.* **16**, 391–401, DOI: 10.5194/nhess-16-391-2016 (2016).

22. Klein, A. & Menezes, J. Beach morphodynamics and profile sequence for a Headland Bay Coast. *J. Coast. Res.* **17**, 812–835 (2001).

23. Almar, R. *et al.* Intertidal beach profile estimation from reflected wave measurements. *Coast. Eng.* **151**, 58–63, DOI: 10.1016/j.coastaleng.2019.05.001 (2019).

24. Price, T., Rutten, J. & Ruessink, B. Coupled behaviour within a double sandbar system. *J. Coast. Res.* 125–129 (2011).

25. Ruessink, B. G., Pape, L. & Turner, I. L. Daily to interannual cross-shore sandbar migration: Observations from a multiple sandbar system. *Cont. Shelf Res.* **29**, 1663–1677, DOI: 10.1016/j.csr.2009.05.011 (2009).

26. Ranasinghe, R., Symonds, G., Black, K. & Holman, R. Morphodynamics of intermediate beaches: a video imaging and numerical modelling study. *Coast. Eng.* **51**, 629–655, DOI: 10.1016/j.coastaleng.2004.07.018 (2004).

27. Turner, I. L. *et al.* A multi-decade dataset of monthly beach profile surveys and inshore wave forcing at Narrabeen, Australia. *Sci. Data* **3**, 160024, DOI: 10.1038/sdata.2016.24 (2016).

28. Short, A. D. Australian Beach System: Nature and Distribution. *J. Coast. Res.* **22**, 11–161 (2006).

29. Wright, L. D., Guza, R. T. & Short, A. D. Dynamics of a high-energy dissipative surf zone. *Mar. Geol.* **45**, 41–62, DOI: 10.1016/0025-3227(82)90179-7 (1982).

30. Shuttleworth, B., Woidt, A., Paparella, T., Herbig, S. & Walker, D. The dynamic behaviour of a river-dominated tidal inlet, River Murray, Australia. *Estuarine, Coast. Shelf Sci.* **64**, 645–657, DOI: 10.1016/j.ecss.2005.04.007 (2005).

31. Short, A. D., Bracs, M. A. & Turner, I. L. Beach oscillation and rotation: local and regional response at three beaches in southeast Australia. *J. Coast. Res.* **70**, 712–717, DOI: 10.2112/SI-120.1 (2014).

32. Gallop, S. L., Bryan, K. R., Coco, G. & Stephens, S. A. Storm-driven changes in rip channel patterns on an embayed beach. *Geomorphology* **127**, 179–188, DOI: 10.1016/j.geomorph.2010.12.014 (2011).

33. McLean, R. F., & Kirk, R. M. Relationships between grain size, size-sorting, and foreshore slope on mixed sand - shingle beaches. *New Zealand J. Geol. Geophys.* **12**, 138–155, DOI: 10.1080/00288306.1969.10420231 (1969). _eprint: https://doi.org/10.1080/00288306.1969.10420231.

34. Pickrill, R. A. & Mitchell, J. S. Ocean wave characteristics around New Zealand. *New Zealand J. Mar. Freshw. Res.* **13**, 501–520, DOI: 10.1080/00288330.1979.9515827 (1979). _eprint: https://doi.org/10.1080/00288330.1979.9515827.

35. Masselink, G. & Short, A. D. The Effect of Tide Range on Beach Morphodynamics and Morphology: A Conceptual Beach Model. *J. Coast. Res.* **9**, 785–800 (1993).

36. Orford, J. D. & Wright, P. What's in a name? — Descriptive or genetic implications of 'ridge and runnel' topography. *Mar. Geol.* **28**, M1–M8, DOI: 10.1016/0025-3227(78)90088-9 (1978).

37. Castelle, B., Bonneton, P., Dupuis, H. & Sénéchal, N. Double bar beach dynamics on the high-energy meso-macrotidal French Aquitanian Coast: A review. *Mar. Geol.* **245**, 141–159, DOI: 10.1016/j.margeo.2007.06.001 (2007).

38. Miles, J., Thorpe, A., Russell, P. & Masselink, G. Observations of bedforms on a dissipative macrotidal beach. *Ocean. Dyn.* **64**, 225–239, DOI: 10.1007/s10236-013-0677-2 (2014).

39. Haxel, J. H. & Holman, R. A. The sediment response of a dissipative beach to variations in wave climate. *Mar. Geol.* **206**, 73–99, DOI: 10.1016/j.margeo.2004.02.005 (2004).

40. Athanasiou, P. *et al.* Global distribution of nearshore slopes with implications for coastal retreat. *Earth Syst. Sci. Data* **11**, 1515–1529, DOI: 10.5194/essd-11-1515-2019 (2019).

41. Forte, M., Birkemeier, W. & Mitchell, J. R. Nearshore survey system evaluation. Tech. Rep., Coastal and Hydraulics Laboratory (U.S.) (2018). DOI: 10.21079/11681/26031.

42. Bishop-Taylor, R., Nanson, R., Sagar, S. & Lymburner, L. Mapping Australia's dynamic coastline at mean sea level using three decades of Landsat imagery. *Remote. Sens. Environ.* **267**, 112734, DOI: 10.1016/j.rse.2021.112734 (2021).

43. Bishop-Taylor, R., Phillips, C., Sagar, S., Newey, V. & Sutterley, T. eo-tides: Tide modelling tools for large-scale satellite Earth observation analysis. *J. Open Source Softw.* **10**, 7786, DOI: 10.21105/joss.07786 (2025).
